# Supplementary material for: Synthesis and Antioxidative Properties of 1,2,3,4-Tetrahydropyridine Derivatives with Different Substituents in 4-Position
Source: Molecules. 2022 Nov 1;27(21):7423. doi: 10.3390/molecules27217423 (PMC9658173; doi:10.3390/molecules27217423)
Supplement: Supplementary file 1 [file molecules-27-07423-s001.zip › molecules-1957293-supplementary.pdf]

Article

Synthesis and antioxidative properties of 1,2,3,4-tetrahydropyridine derivatives with different substituents in 4-position

Daniele Aiello[1,2], Hendrik Jonas[1,3], Anna Carbone[1,4], Daniela Carbone[1], Camilla Pecoraro[1], Luisa Tesoriere[1], Jens Köhler[3], Bernhard Wünsch[3], Patrizia Diana[1,*]

[1] Dipartimento di Scienze e Tecnologie Biologiche Chimiche e Farmaceutiche (STEBICEF), Università degli Studi di Palermo, Via Archirafi 32, 90123 Palermo, Italy
[2] Dipartimento di Scienze della Vita, Università degli Studi di Modena e Reggio Emilia, Via Campi 103, 41125 Modena, Italy
[3] Westfälische Wilhelms-Universität Münster, Institut für Pharmazeutische und Medizinische Chemie, Corrensstraße 48, D-48149 Münster, Germany
[4] Dipartimento di Farmacia, Università degli Studi di Genova, Viale Benedetto XV 3, 16132 Genova, Italy
* Correspondence: patrizia.diana@unipa.it

Citation: Lastname, F.; Lastname, F.; Lastname, F. Title. *Molecules* **2022**, *27*, 7423. https://doi.org/10.3390/molecules27217423

Academic Editor: Firstname Lastname

Received: date
Accepted: date
Published: date

Publisher's Note: MDPI stays neutral with regard to jurisdictional claims in published maps and institutional affiliations.

Copyright: © 2022 by the authors. Submitted for possible open access publication under the terms and conditions of the Creative Commons Attribution (CC BY) license (https://creativecommons.org/licenses/by/4.0/).

Content **Page**

1.0 Folin Cicalteau assay S2
2.0 NMR spectra S3

1. **Folin-Ciocalteu assay**

1.1Preliminary procedures

- Prepare a solution of Na_2CO_3 2 % (w/v) by weighting 2 g of Na_2CO_3 and dissolve in 100 mL of distilled water.
- Dilute FCR with distilled water to obtain a solution 50 % (v/v).
- Prepare a solution of Gallic acid (standard) 6 mM by weighting 1 mg of gallic acid (PM = 170,12) and solubilize in 1 mL of distilled water.

The quantities of solution of gallic acid 6 nM used are:

- 10μL (10μg) + 90 μL di H_2O
- 30μL (30μg) + 70 μL di H_2O
- 50μL (50μg) + 50 μL di H_2O
- 70μL (70μg) + 30 μL di H_2O

The final volumes of the sample and standard must be equal to 100 μL.

1.2 General procedure

- In each tube add the exact volume of distilled water to reach 100 μL then add sample or standard.
- Prepare the standard with 100 μL.
- Add 3 mL of 2% Na_2CO_3 (w/v) solution.
- Add 100 μLof FCR solution 50 % (v/v).
- Stirring and incubation at RT in dark place for 60 min
- Measure the optical density with an UV-spectrophotometer at 765 nm.
- Create the curve for data analysis
- Express the results in mg GAE/g sample.

1.3 Determination of the TPC of compounds 21

The samples were solubilized in DMSO to obtain a 100 mM solution. For each sample two aliquots of 1 μL and 100 μL were used and the volumes were adjusted to 100 μL with distilled water. To the samples 3 mL of 2% Na_2CO_3 (w/v) solution were added followed by addition of 100 μL of FCR. The samples were incubated for 60 min in a dark place at RT. After 60 min no color change was observed indicating the absence of antioxidant activity.

2. NMR spectra

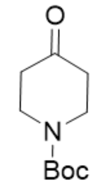

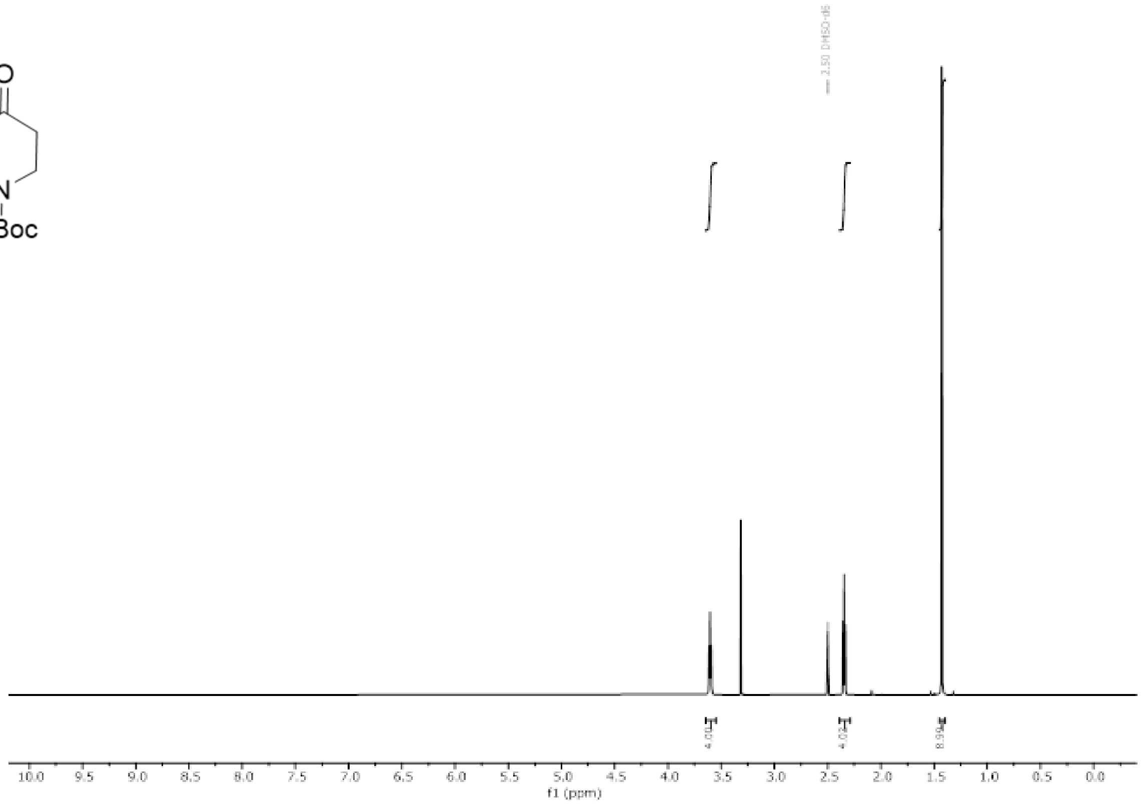

^{1}H NMR spectrum (DMSO-d_6) of **18**

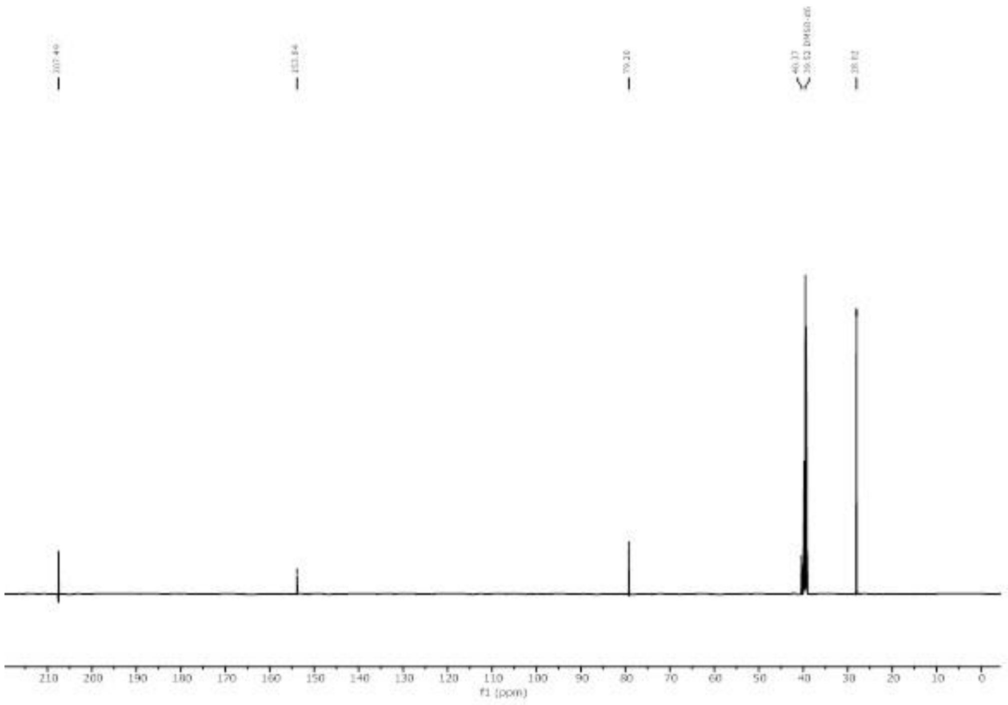

^{13}C NMR spectrum (DMSO-d_6) of **18**

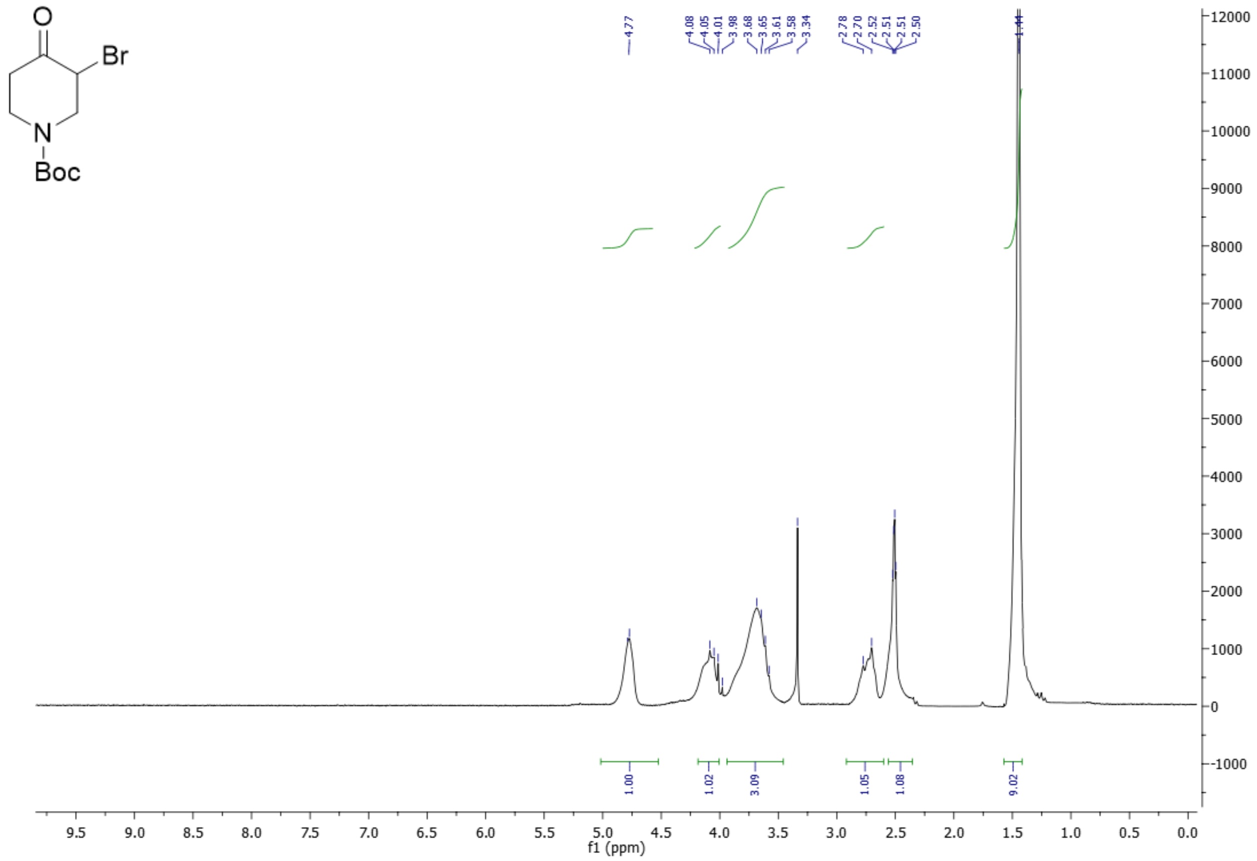

^{1}H NMR spectrum (DMSO-d_6) of **17.**

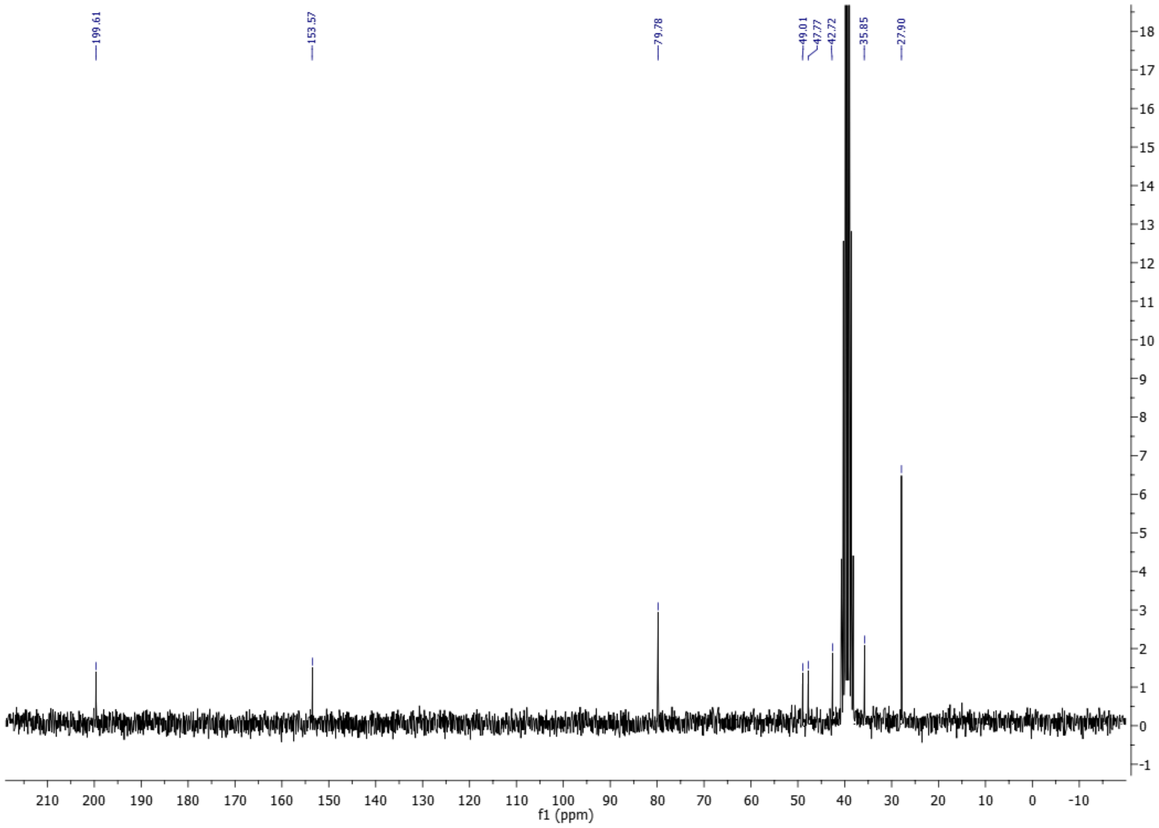

^{13}C NMR spectrum (DMSO-d_6) of **17**

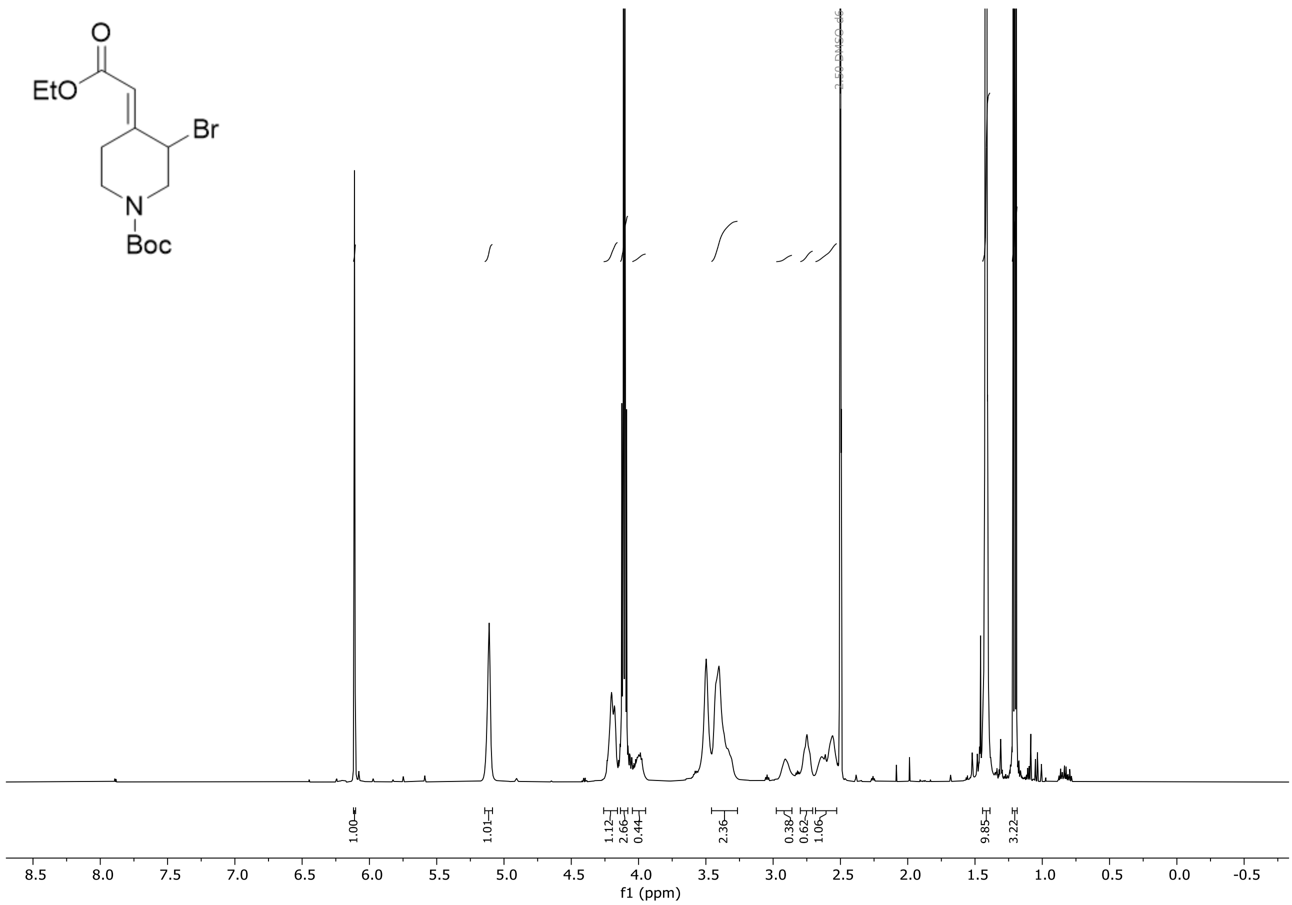

^{1}H NMR spectrum (DMSO-d_6) of **16.**

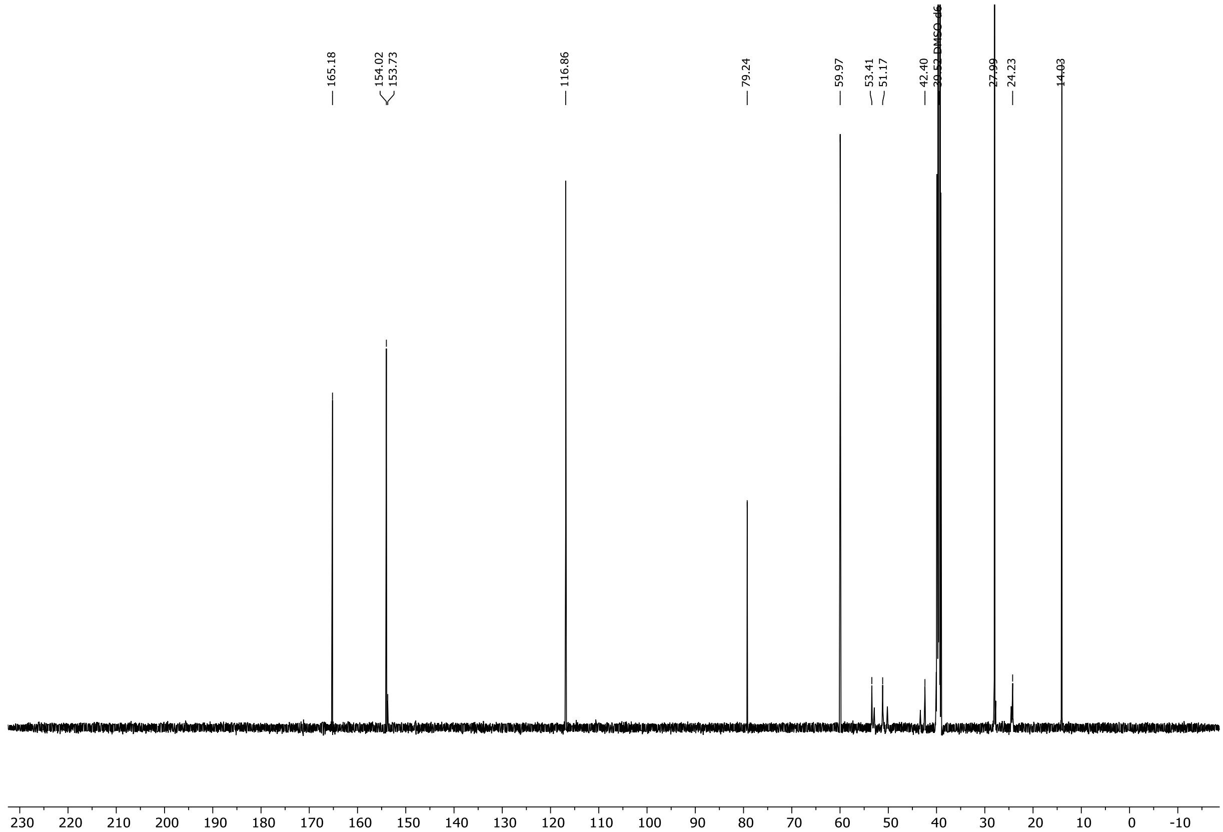

^{13}C NMR spectrum (DMSO-d_6) of **16.**

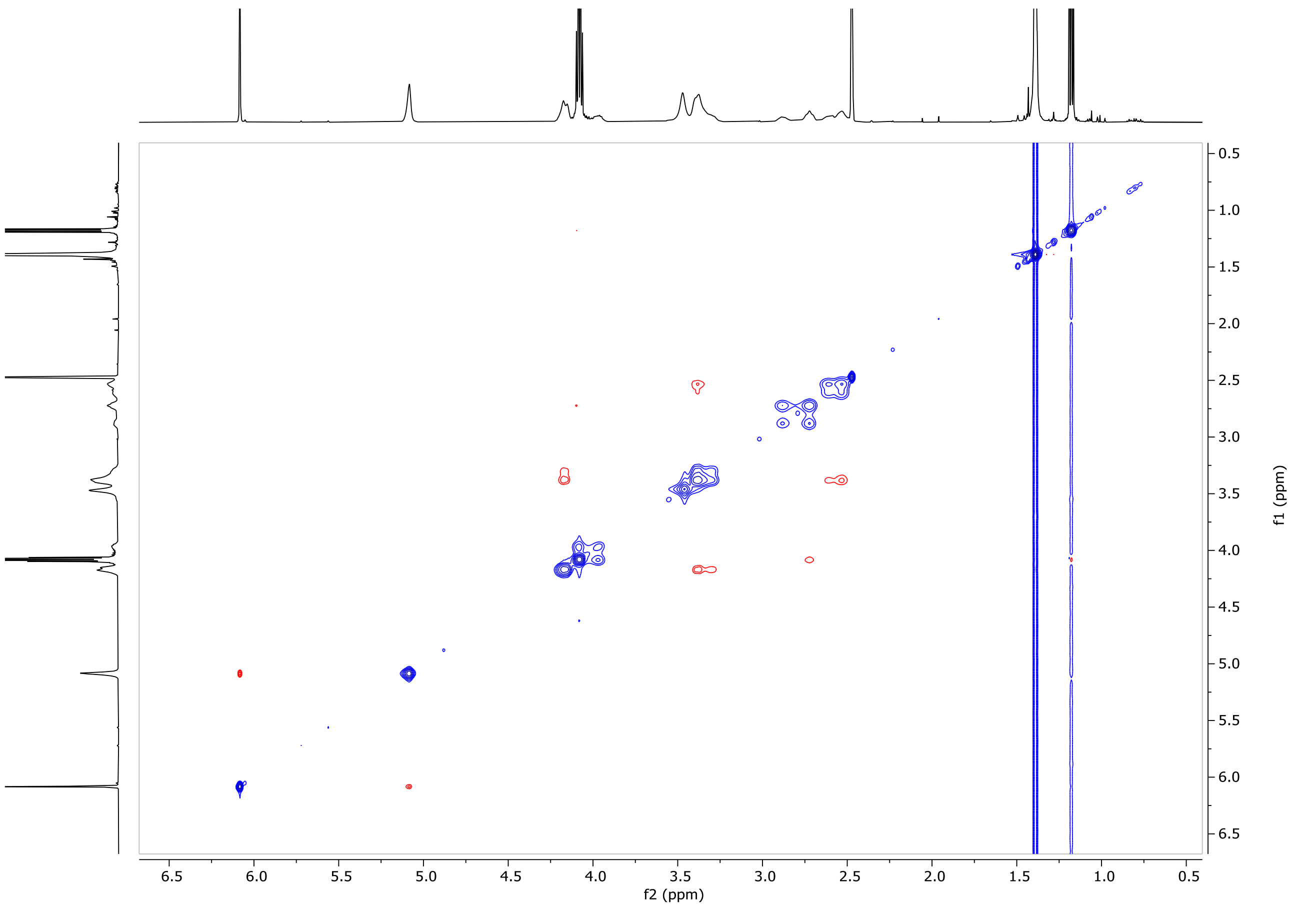

NOESY spectrum of **16**

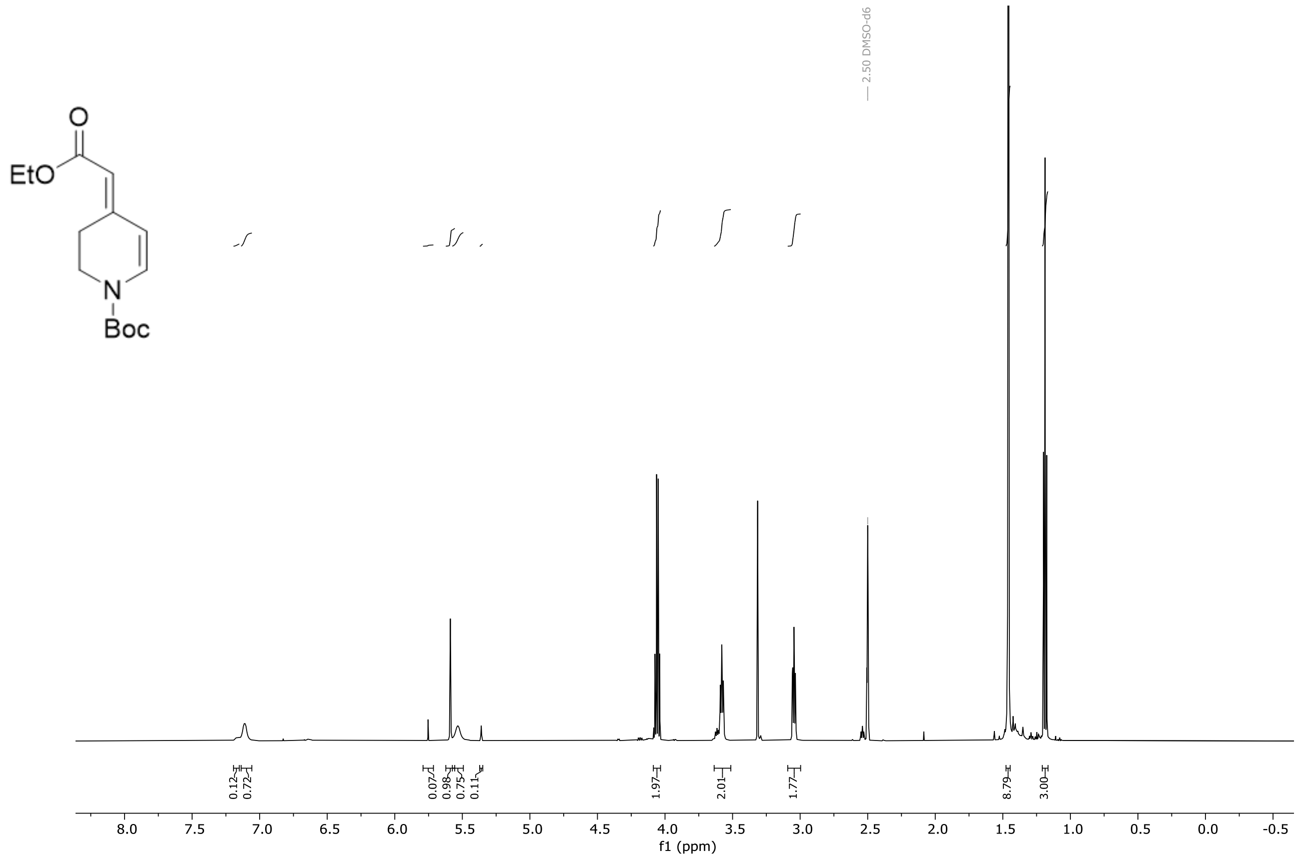

^{1}H NMR spectrum (DMSO-d_6) of **15.**

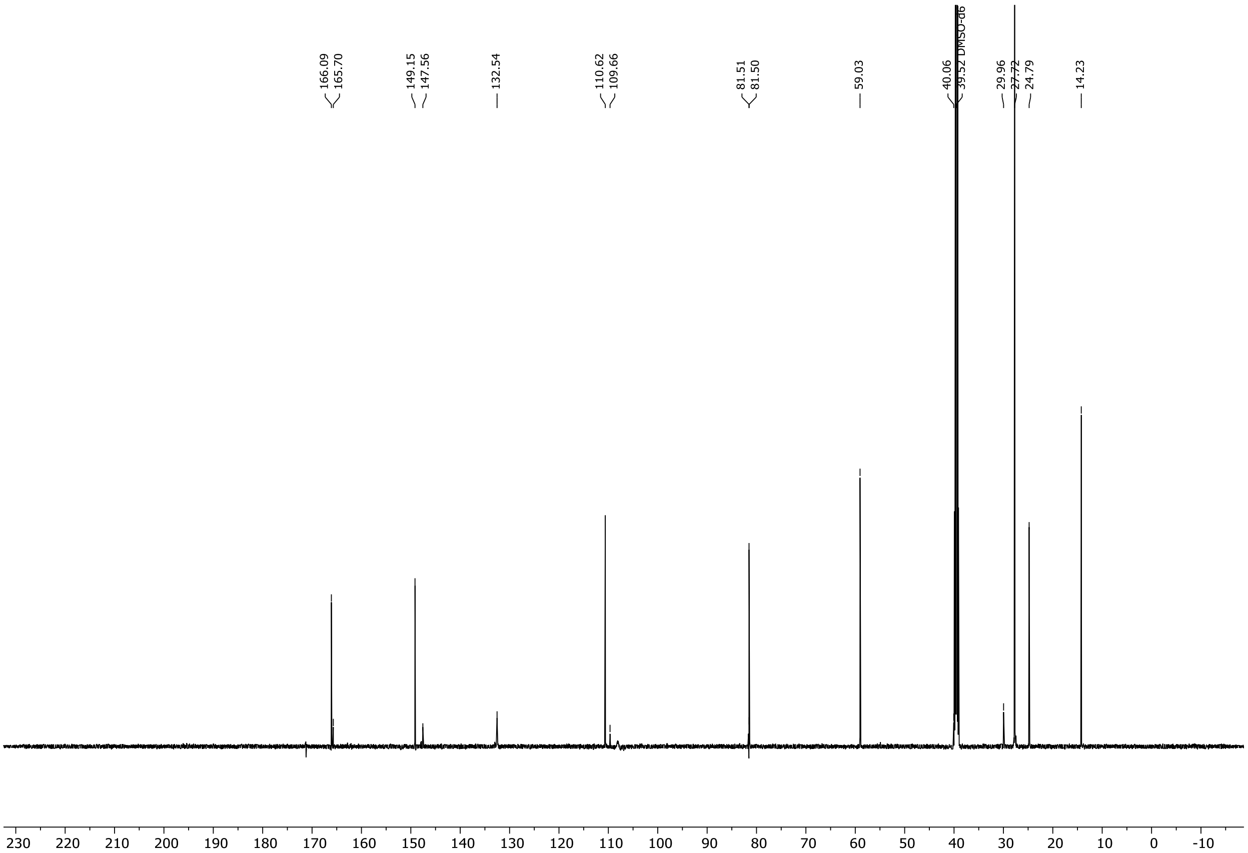

^{13}C NMR spectrum (DMSO-d_6) of **15.**

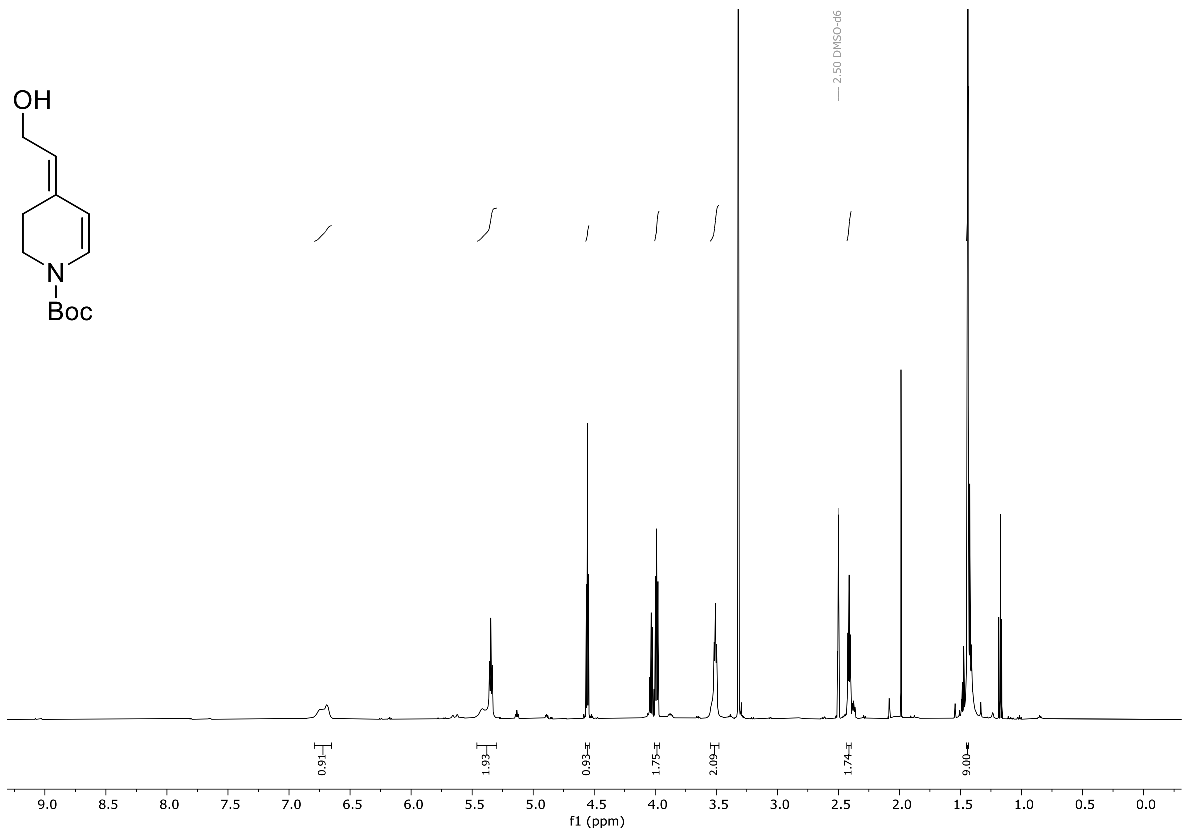

^{1}H NMR spectrum (DMSO-d$_6$) of **20.**

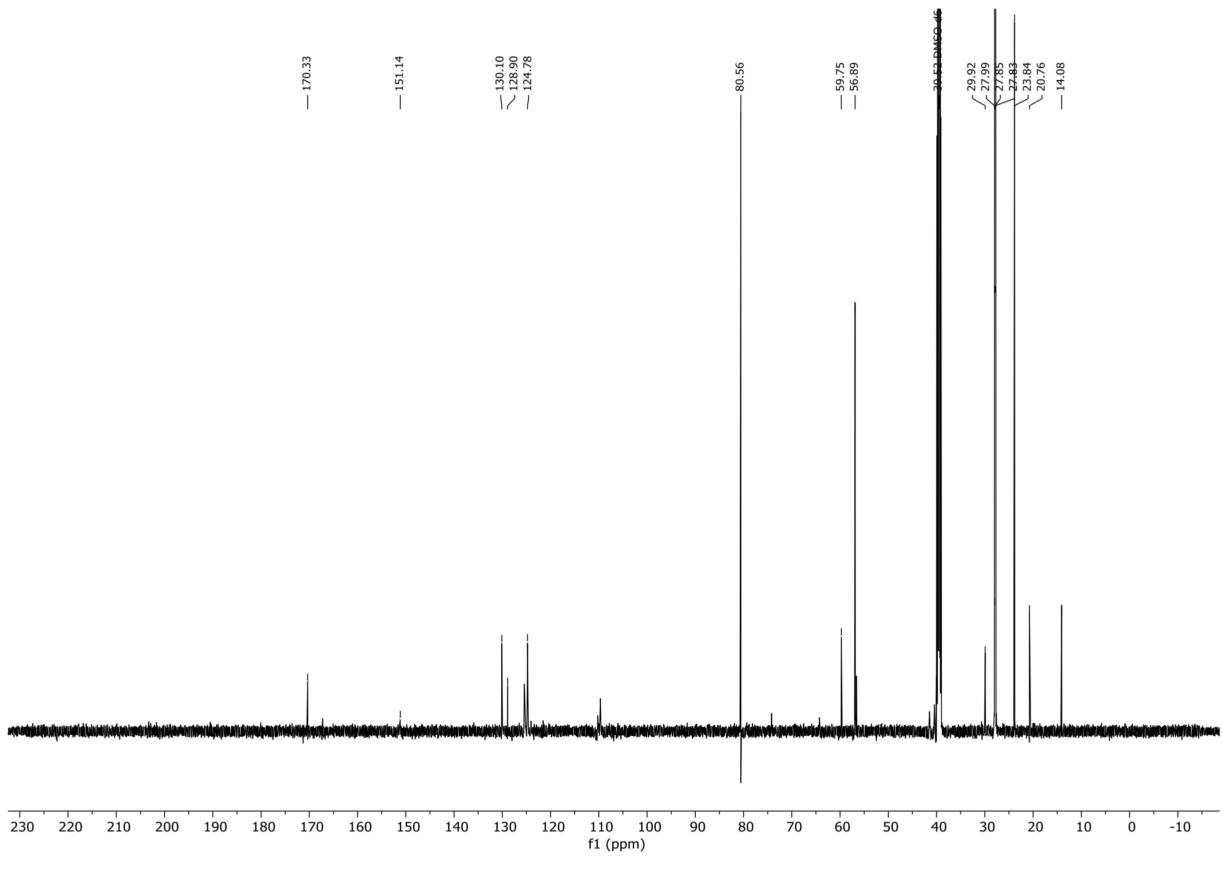

^{13}C NMR spectrum (DMSO-d$_6$) of **20.**

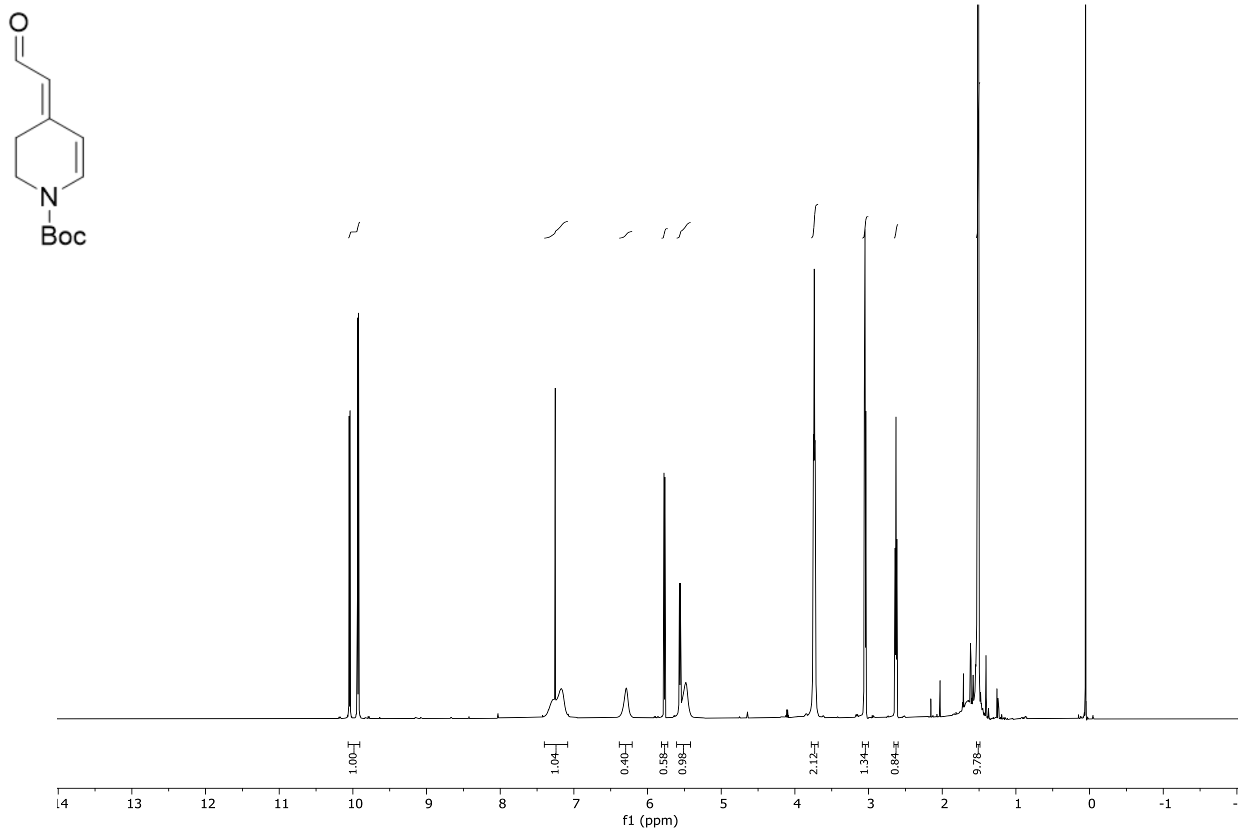

^{1}H NMR spectrum ($CDCl_3$) of **21.**

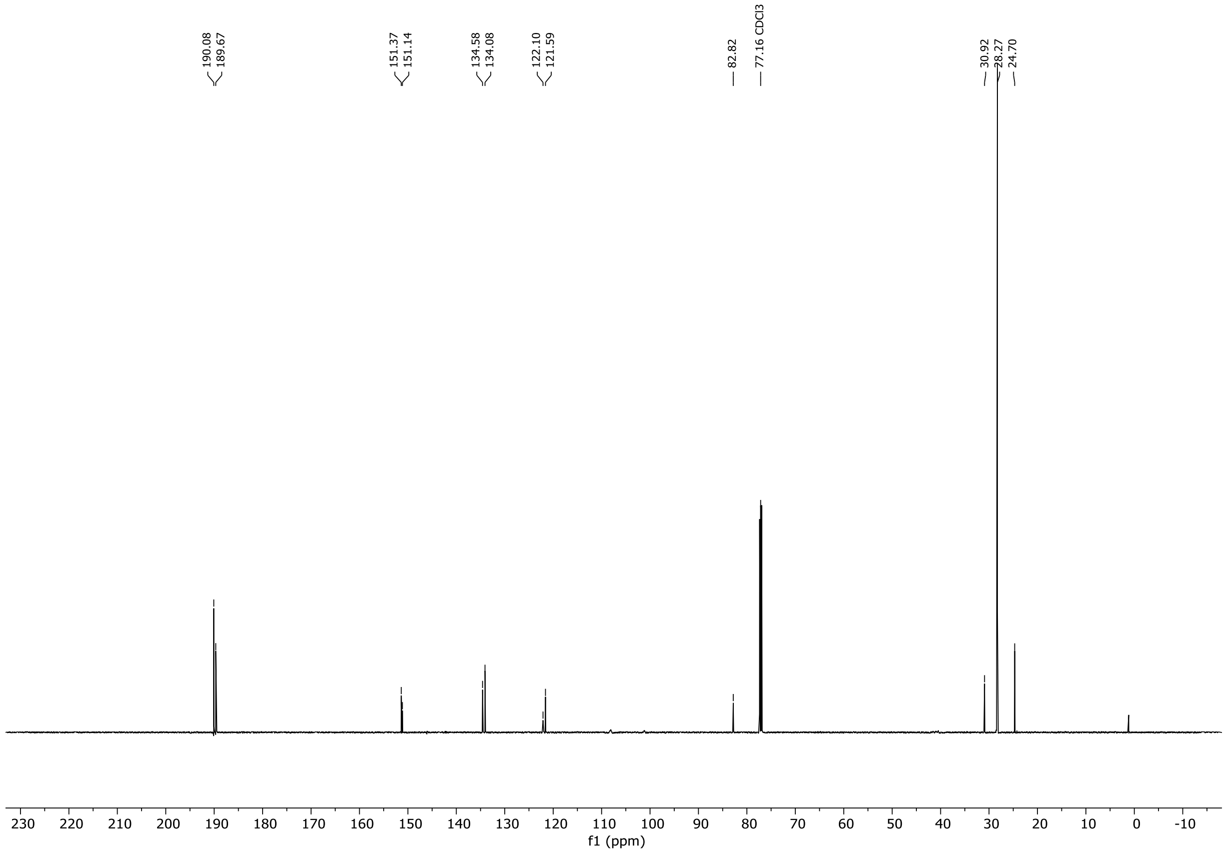

^{13}C NMR spectrum ($CDCl_3$) of **21.**

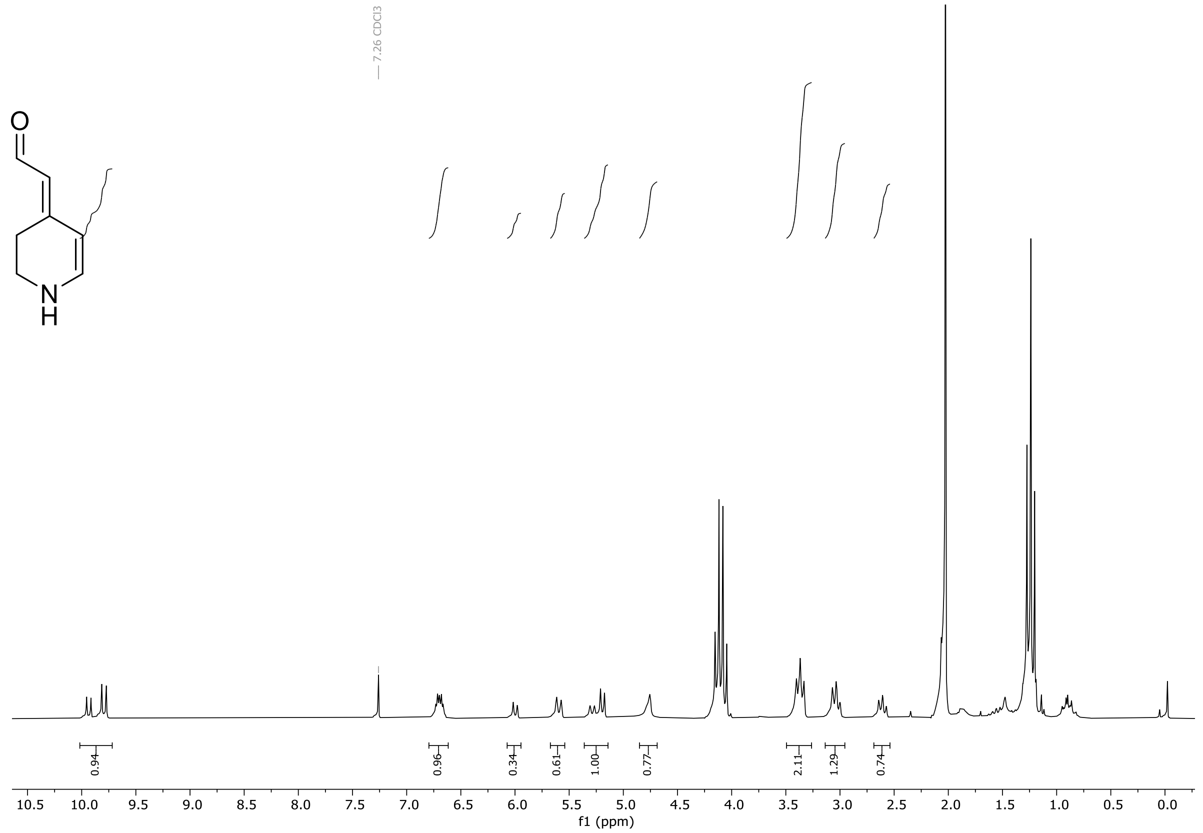

^{1}H NMR spectrum (CDCl$_3$) of **13.**

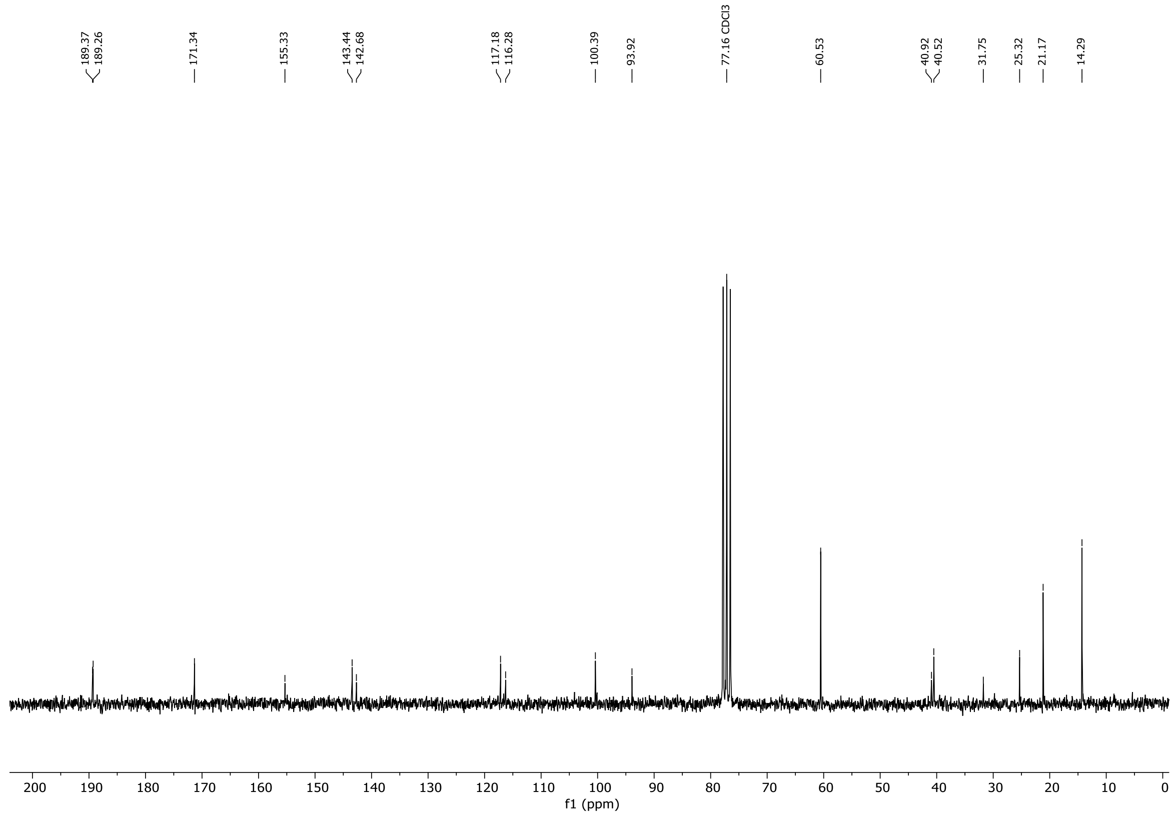

^{13}C NMR spectrum (CDCl$_3$) of **13**.

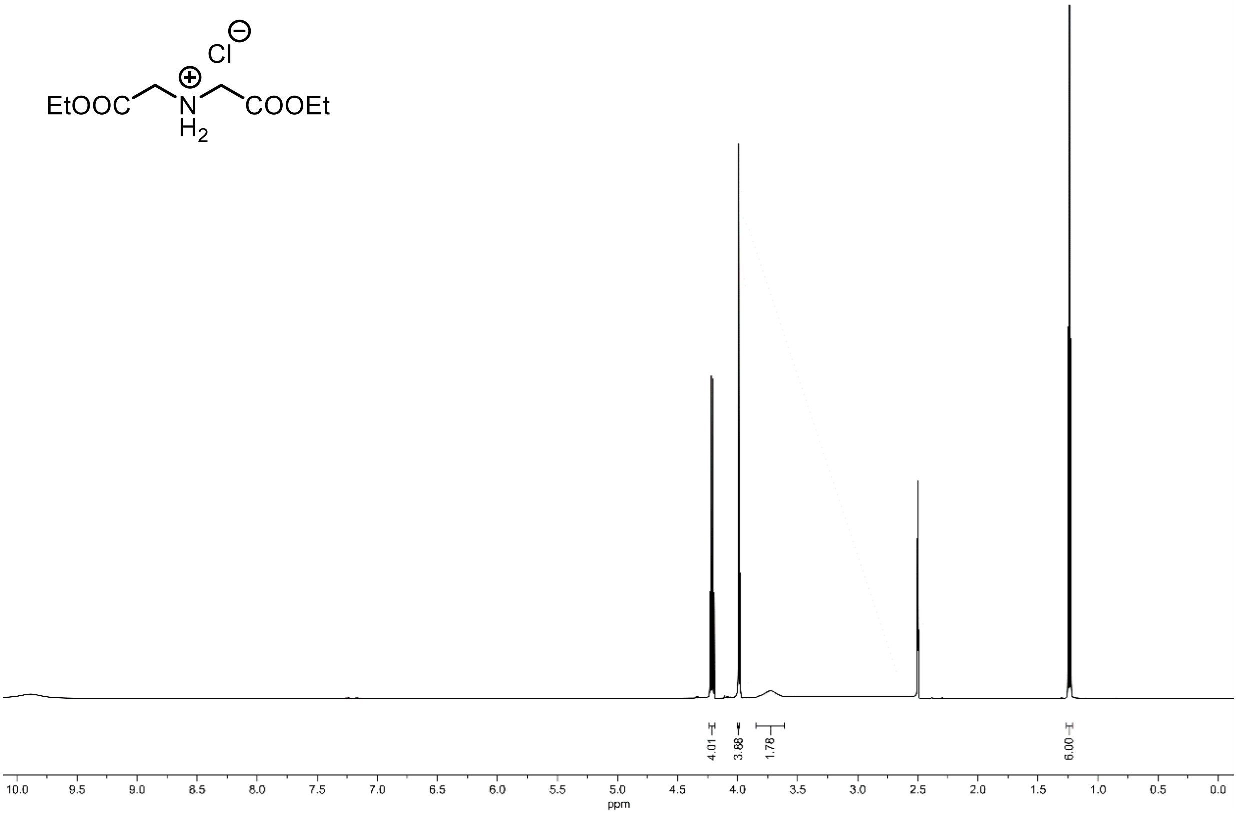

^{1}H NMR spectrum (DMSO-d_6) of **28**.

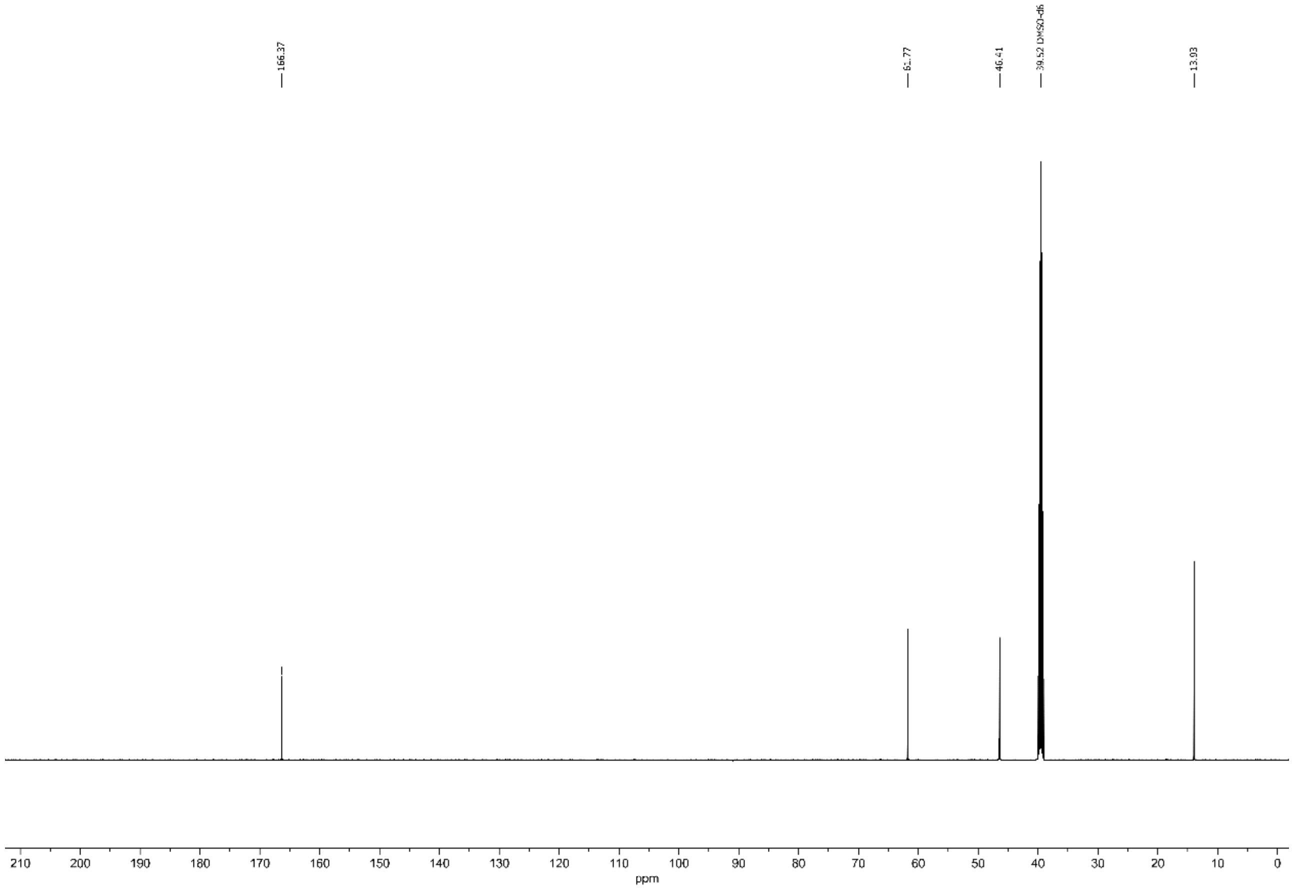

^{13}C NMR spectrum (DMSO-d_6) of **28**.

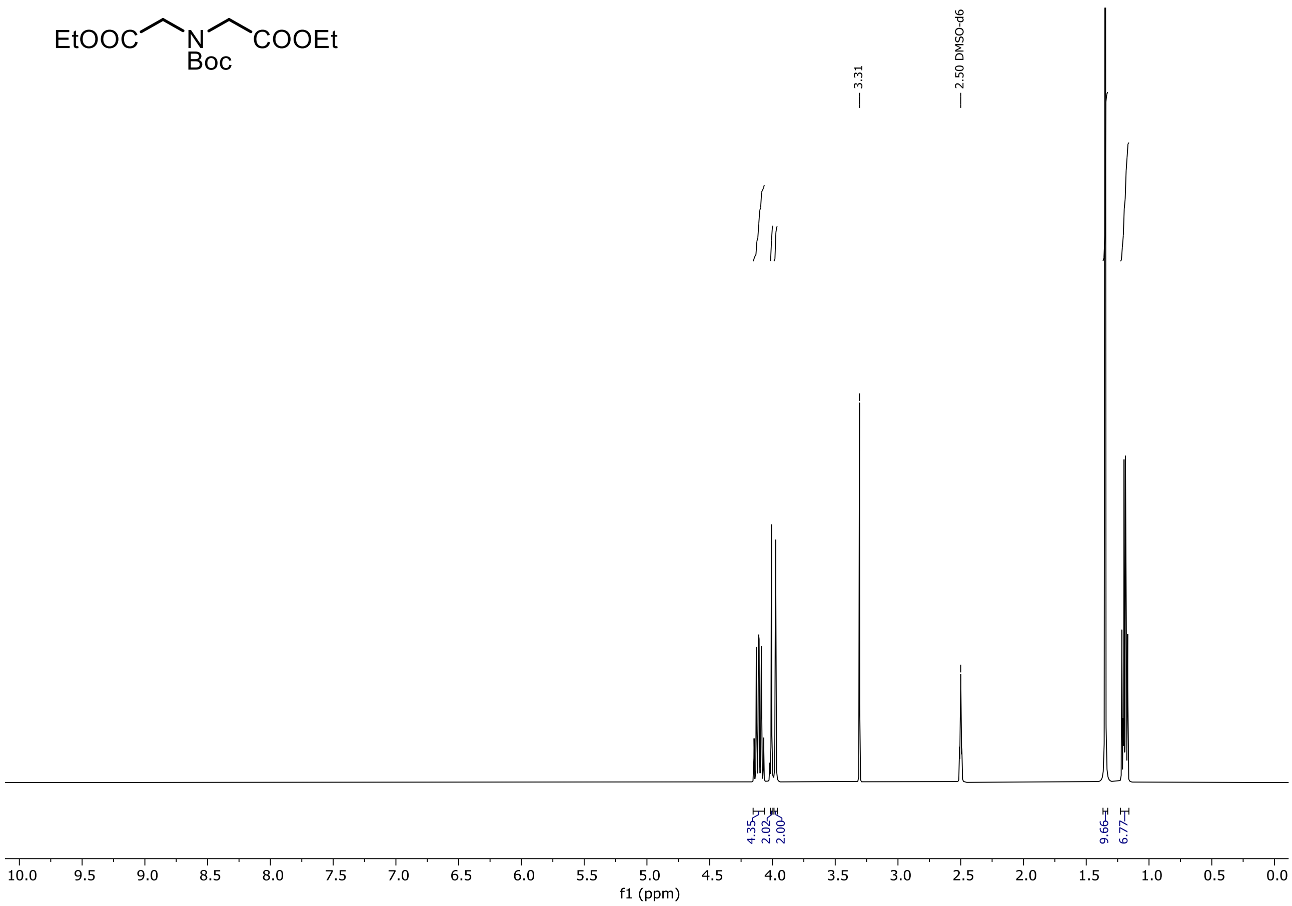

^1H NMR spectrum (DMSO-d_6) of **25**. Contains traces of H_2O.

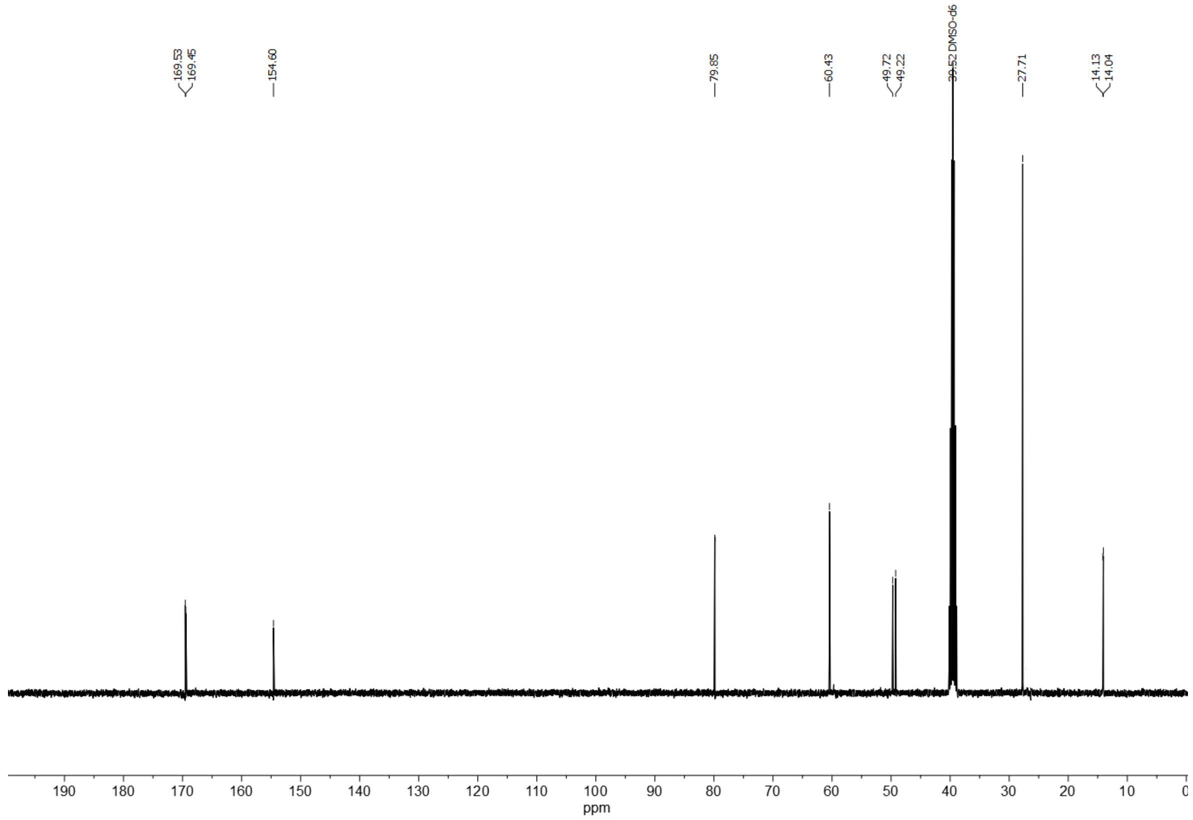

^{13}C NMR spectrum (DMSO-d_6) of **25**.

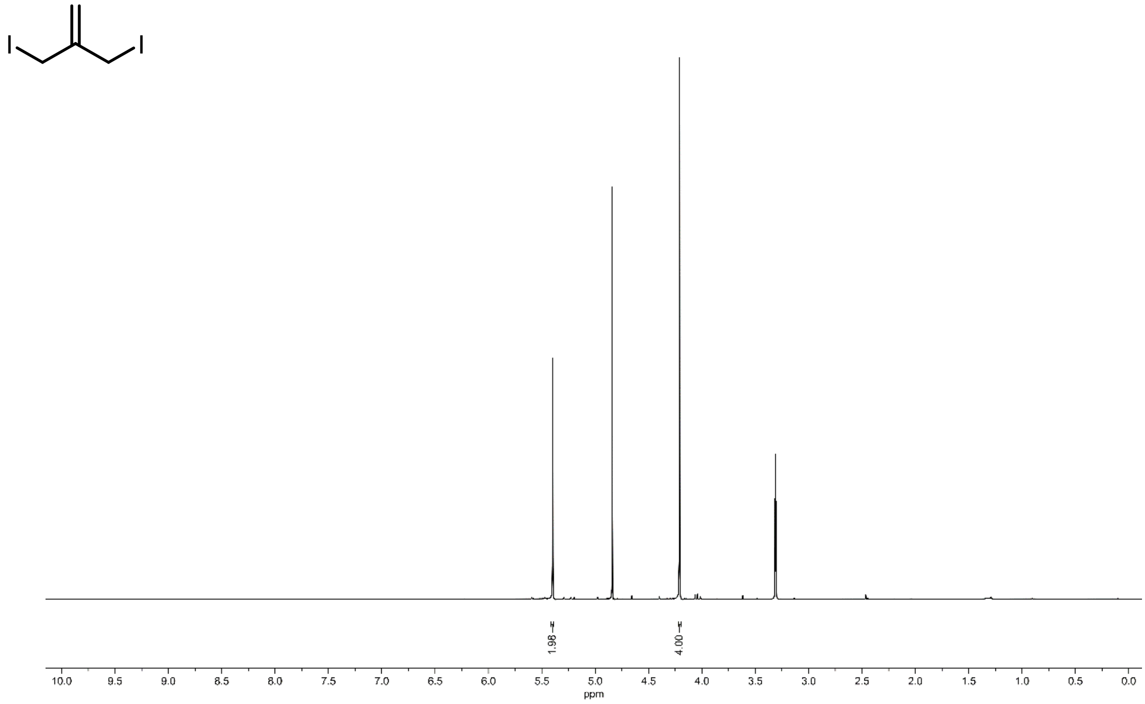

^{1}H NMR spectrum (CD_3OD) of **29**.

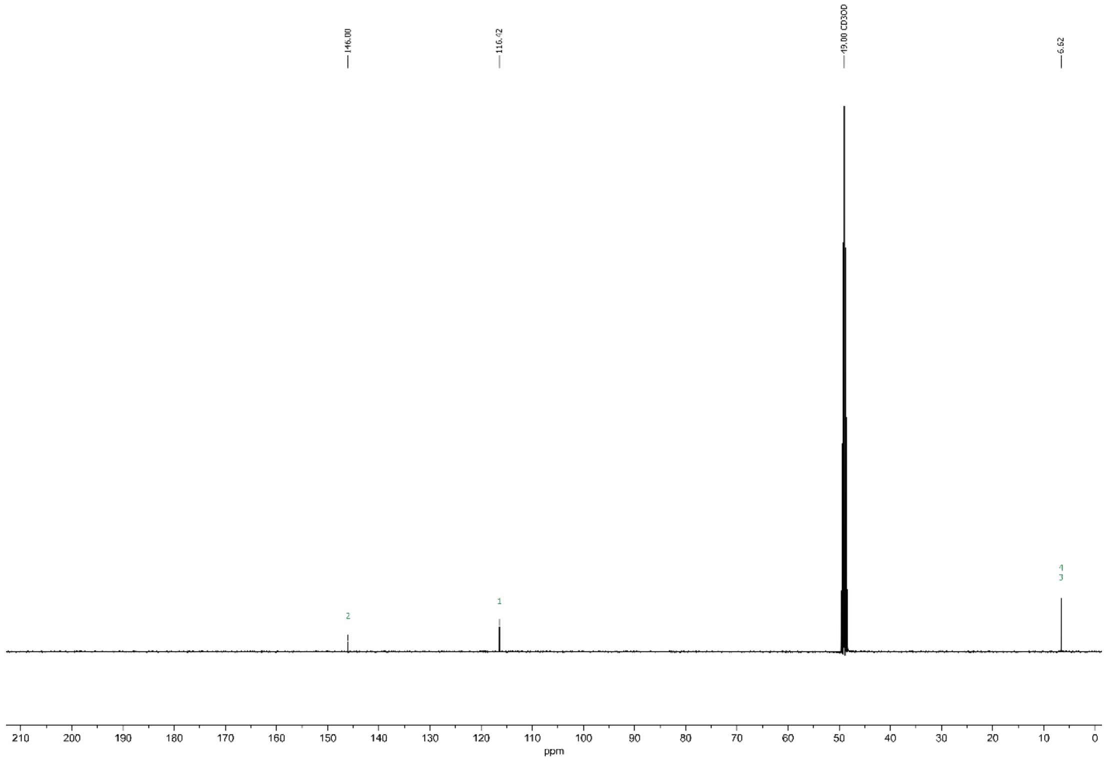

^{13}C NMR spectrum (CD_3OD) of **29**.

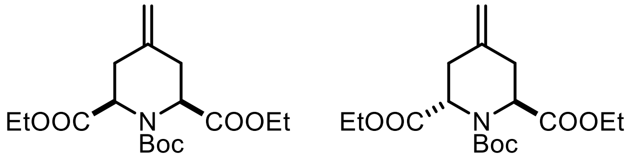

cis-**24** *trans*-**24**

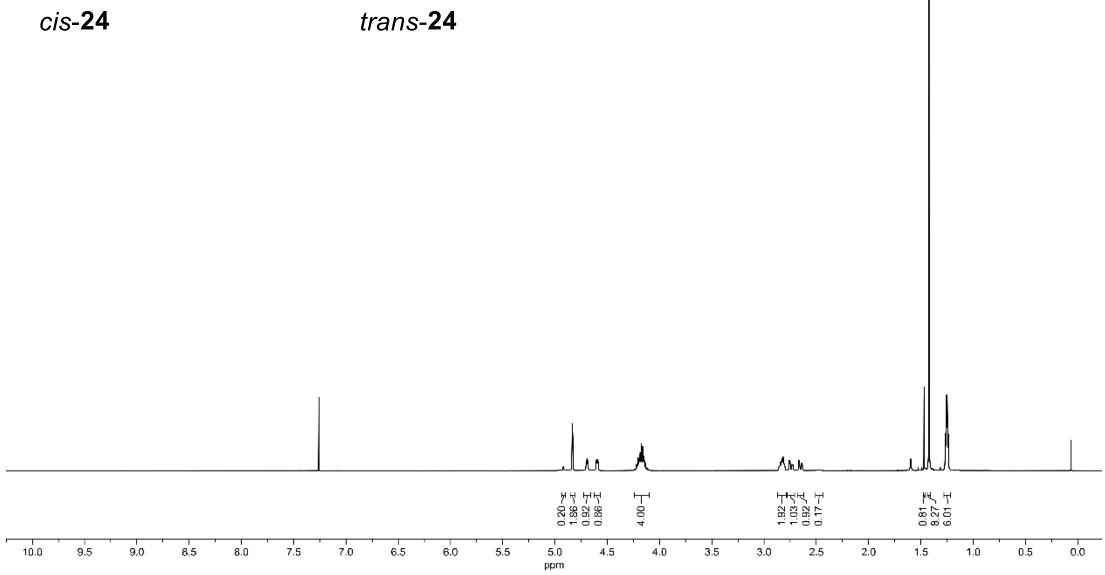

^{1}H NMR spectrum ($CDCl_3$) of **24**. The diastereomers were obtained in a 9:1 ratio.

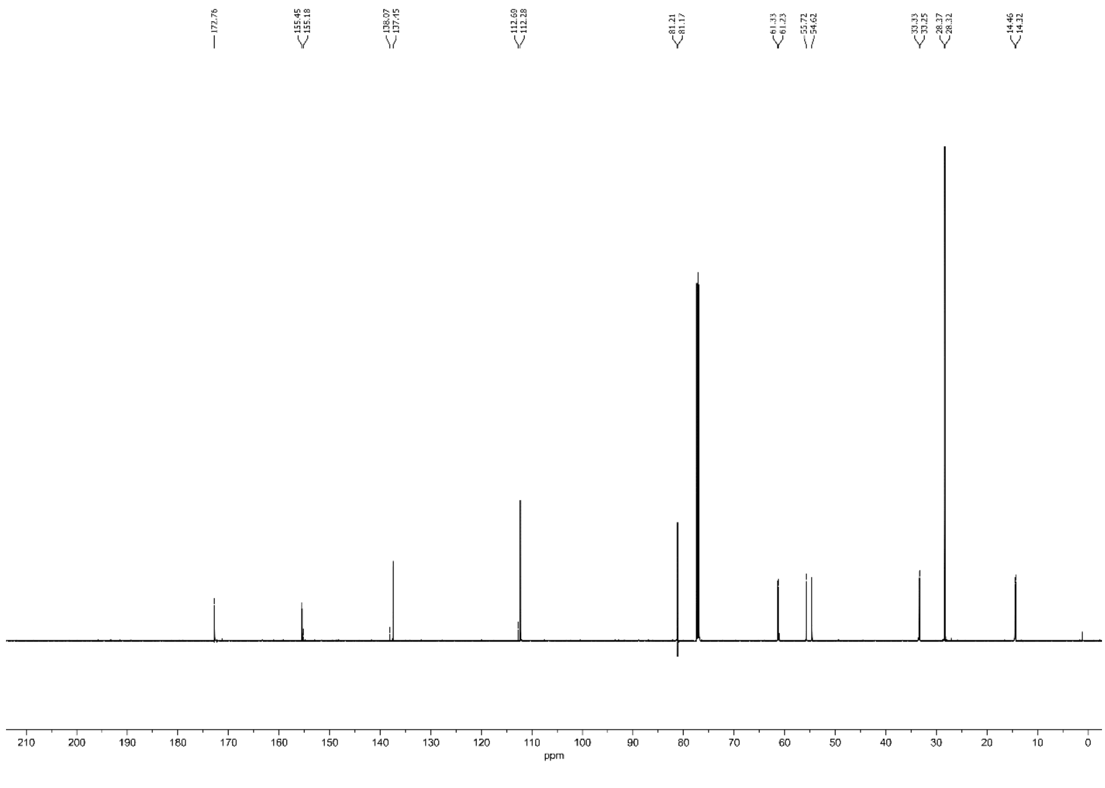

^{13}C NMR spectrum ($CDCl_3$) of **24**. The diastereomers were obtained in a 9:1 ratio.

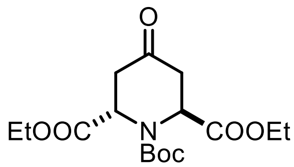

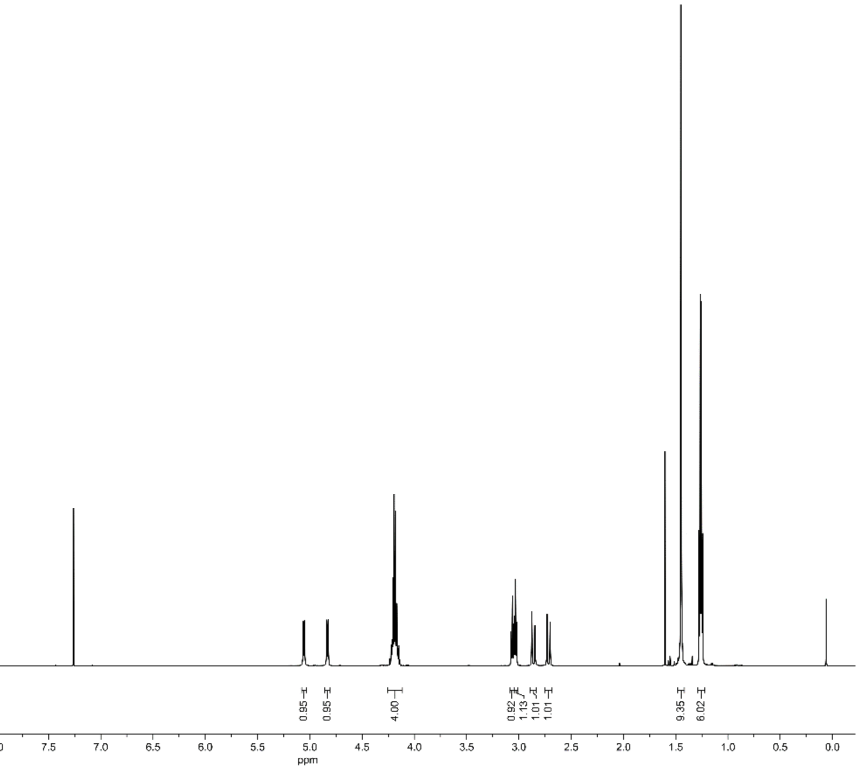

^{1}H NMR spectrum ($CDCl_3$) of **30**.

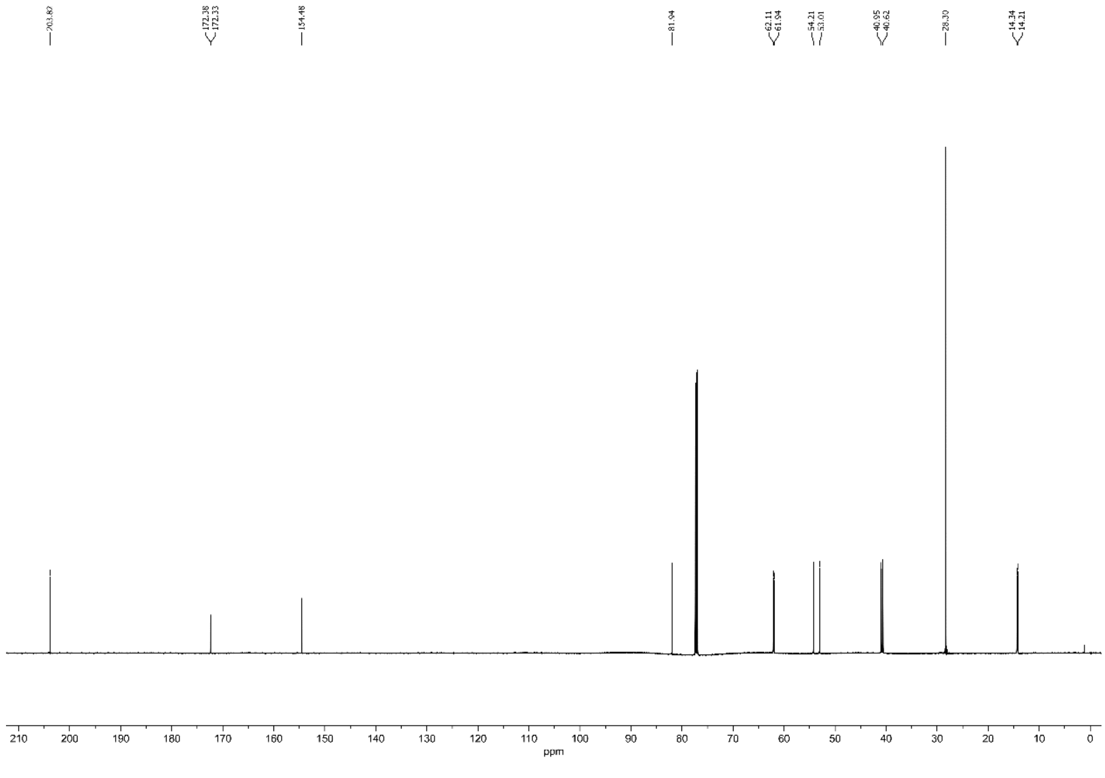

^{13}C NMR spectrum ($CDCl_3$) of **30**.

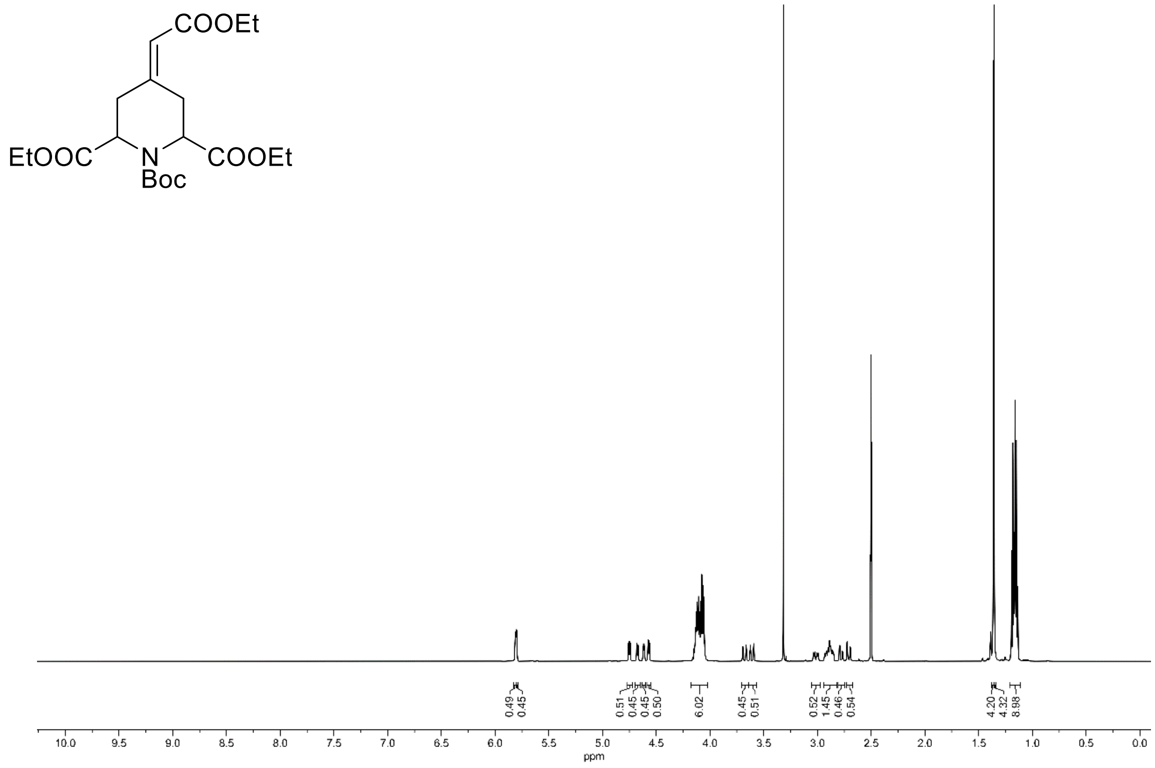

^{1}H NMR spectrum (DMSO-d_6) of **31**.

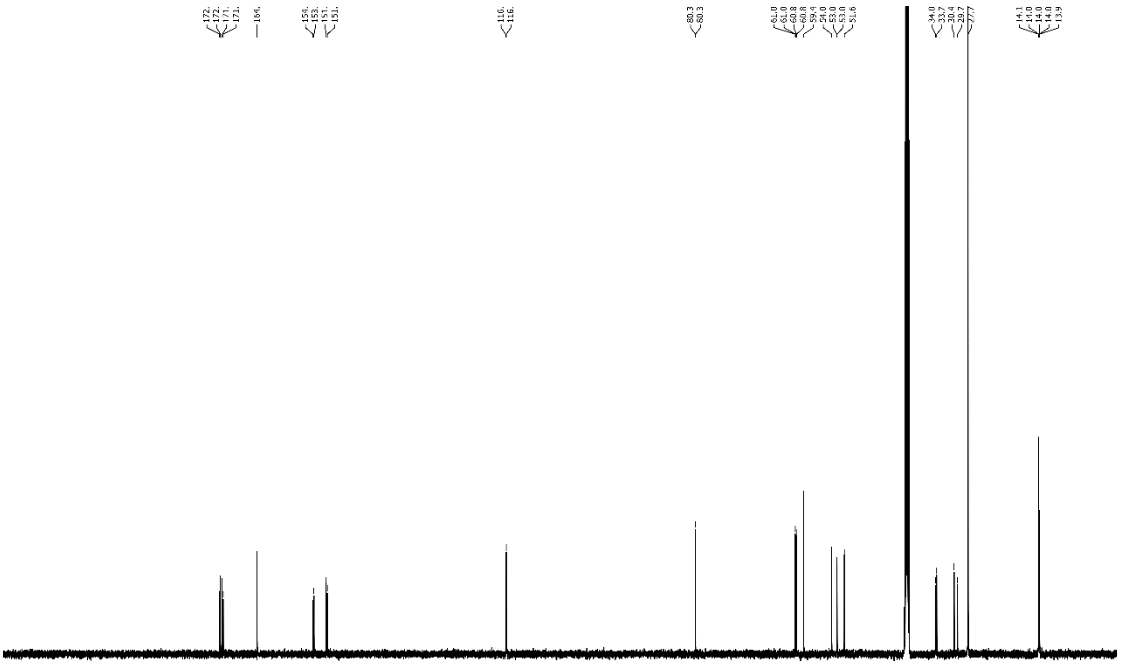

^{13}C NMR spectrum (DMSO-d_6) of **31**.

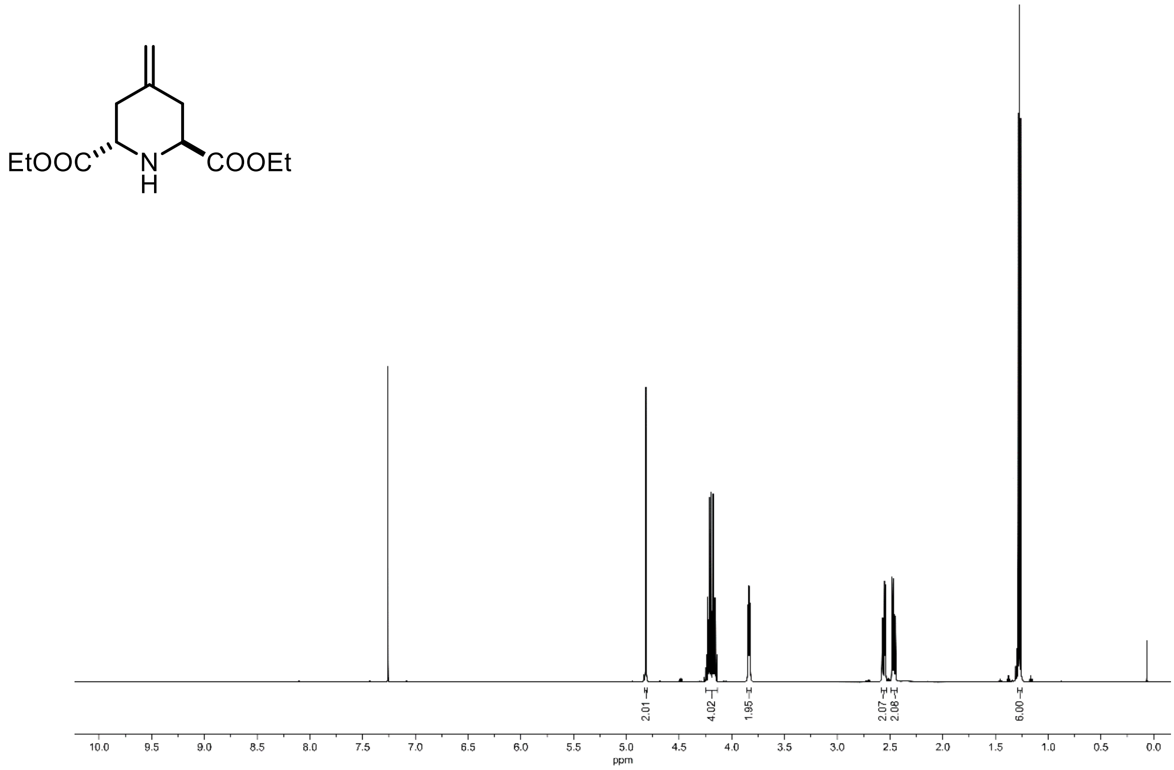

^{1}H NMR spectrum ($CDCl_3$) of **32**.

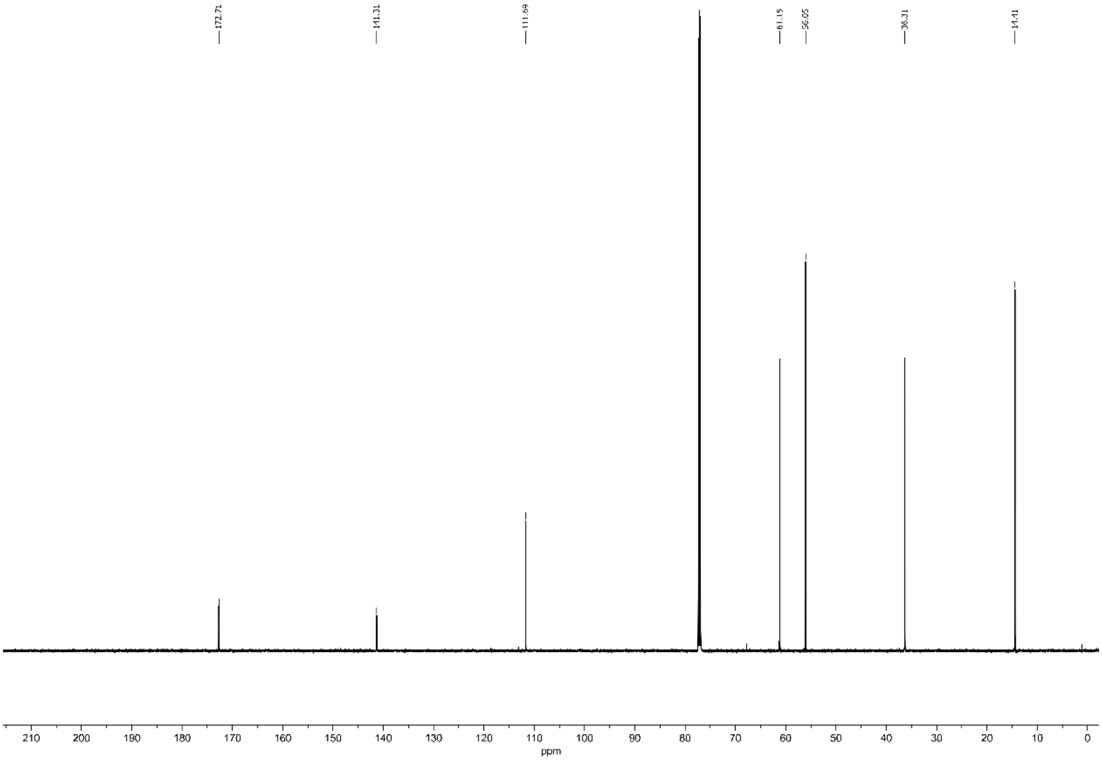

^{13}C NMR spectrum ($CDCl_3$) of **32**.

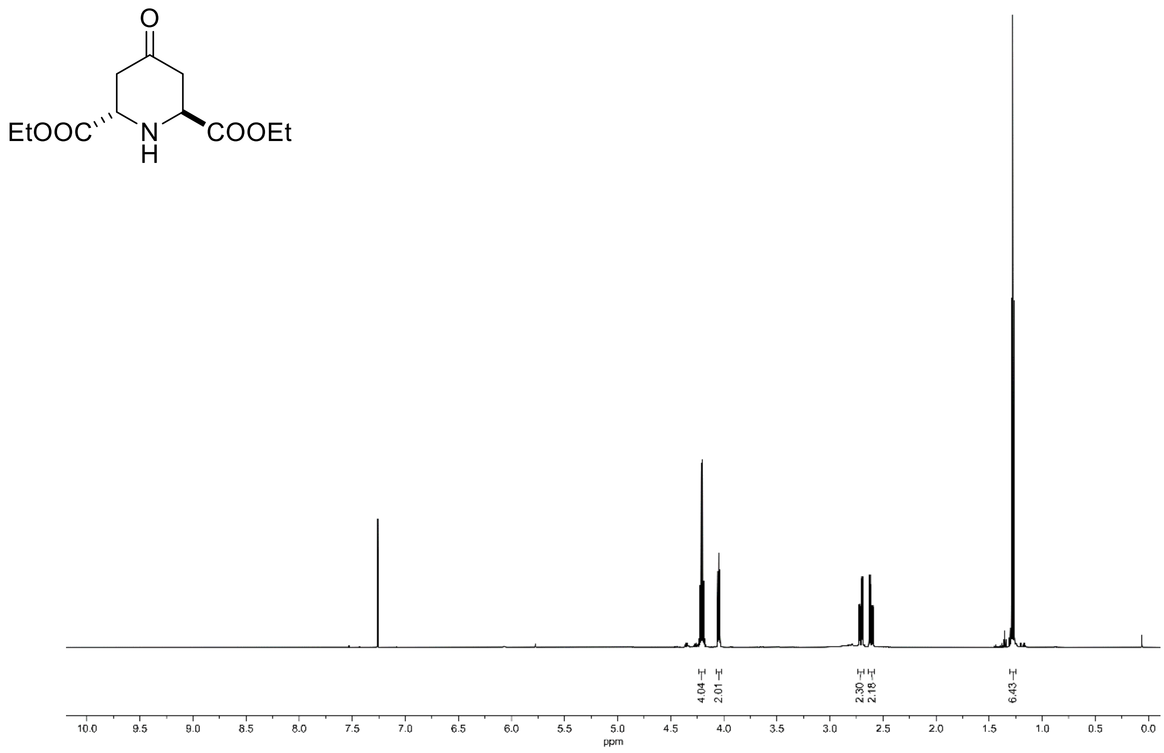

^{1}H NMR spectrum ($CDCl_3$) of **33**.

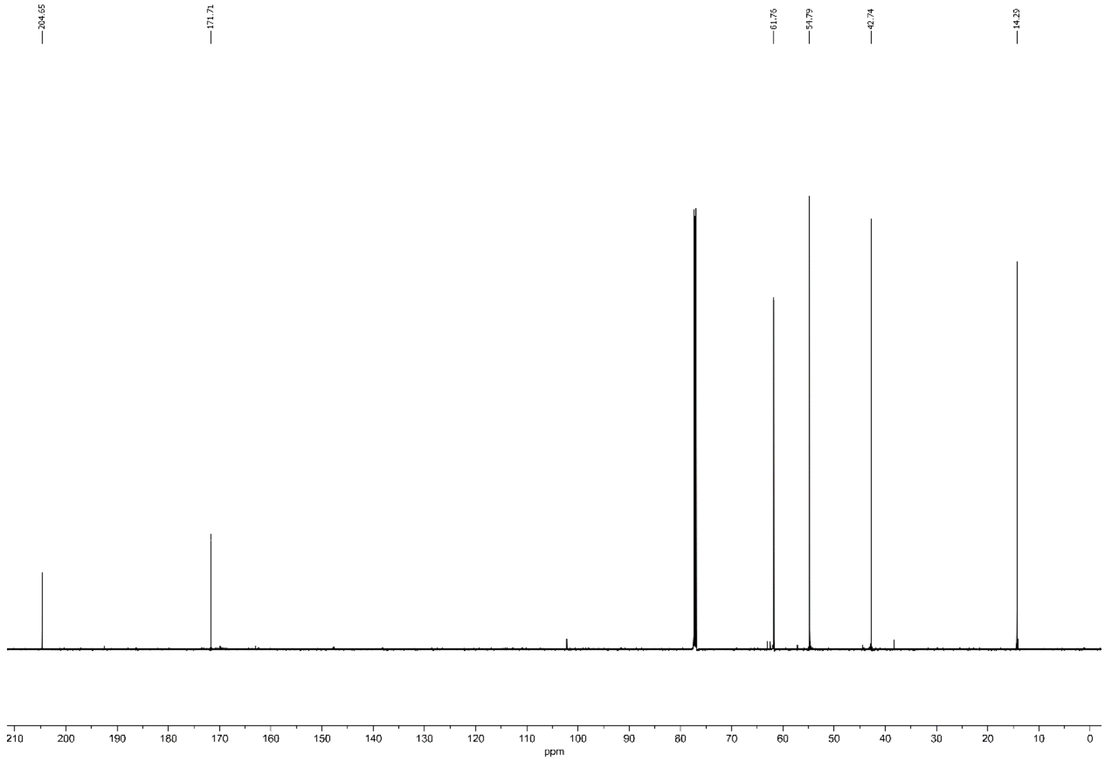

^{13}C NMR spectrum ($CDCl_3$) of **33**.

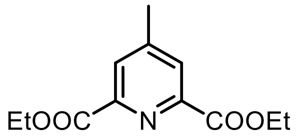

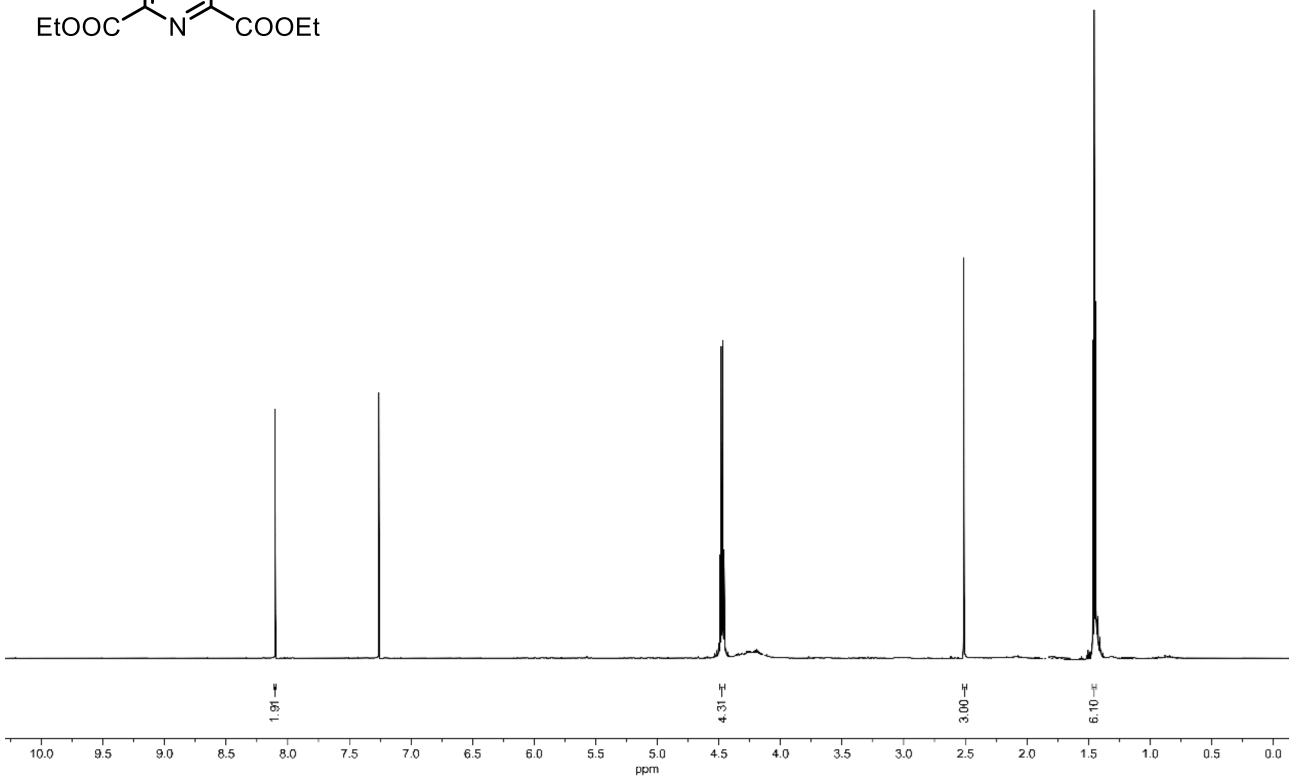

^{1}H NMR spectrum ($CDCl_3$) of **38**.

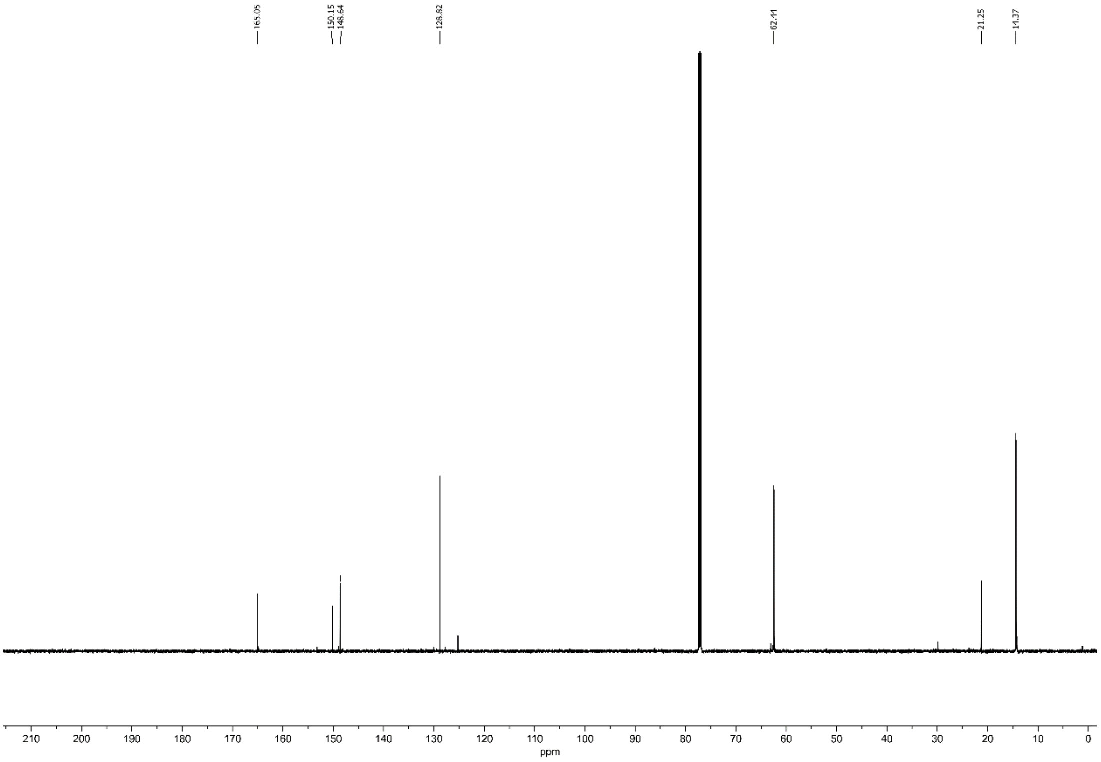

^{13}C NMR spectrum ($CDCl_3$) of **38**.

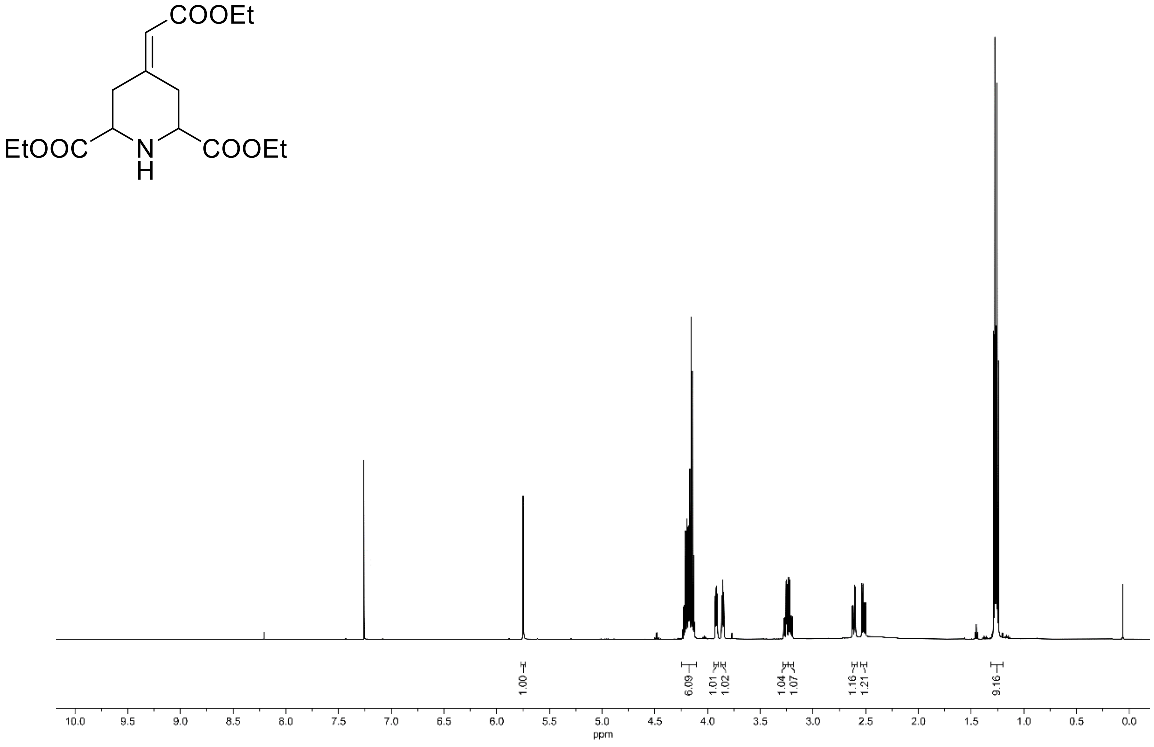

^{1}H NMR spectrum ($CDCl_3$) of **34**.

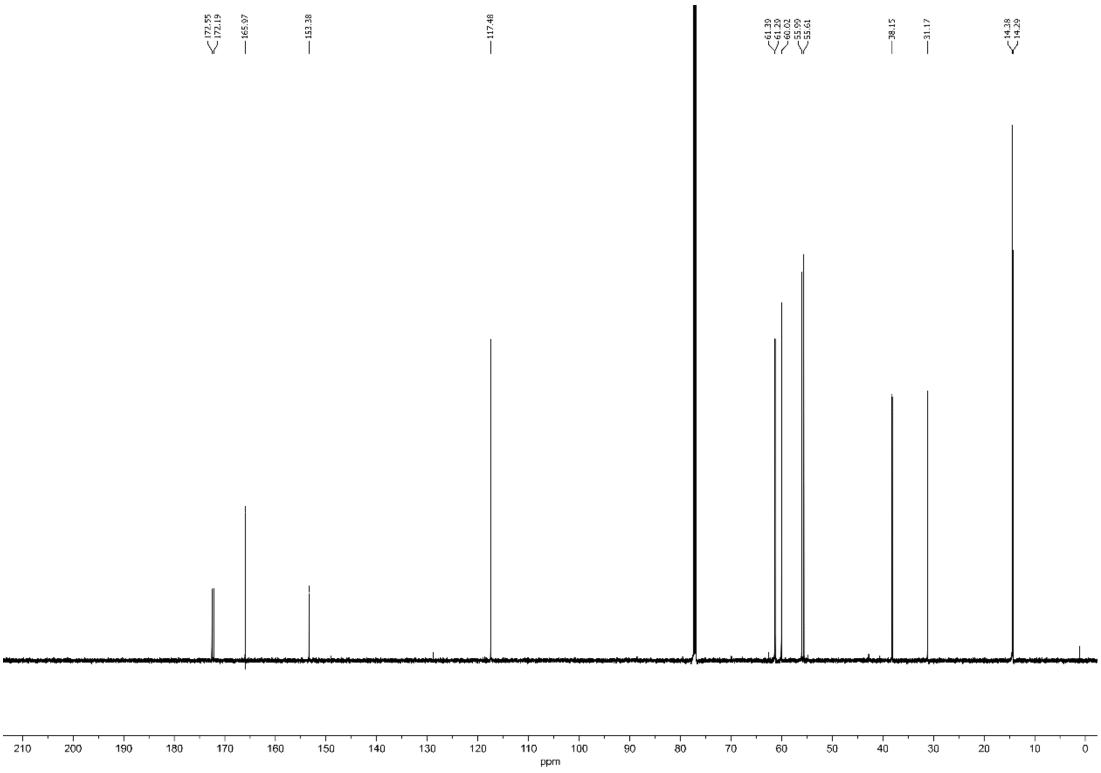

^{13}C NMR spectrum ($CDCl_3$) of **34**.

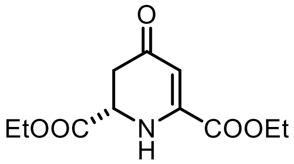

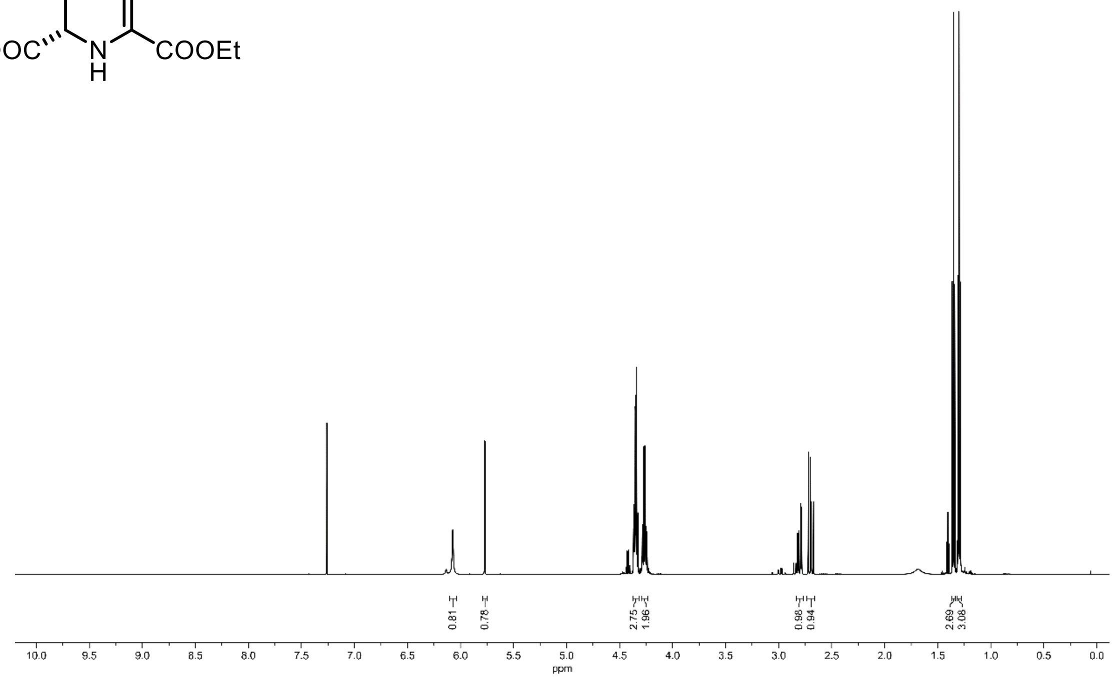

¹H NMR spectrum ($CDCl_3$) of **36**.

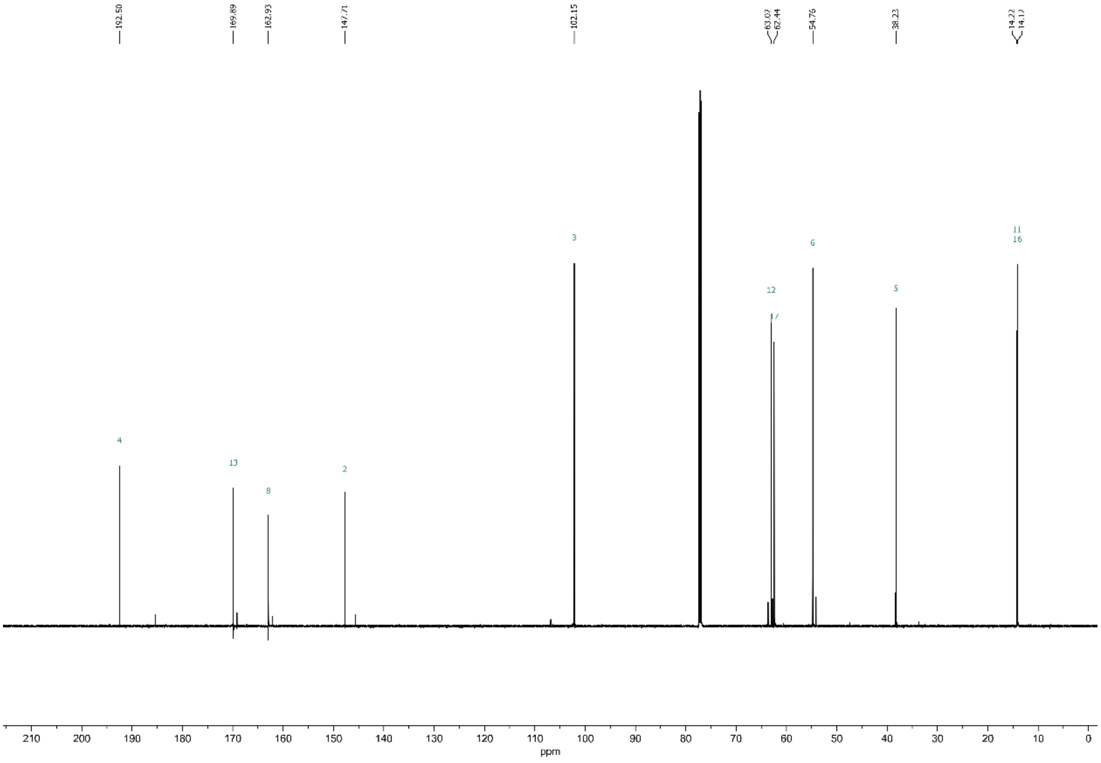

¹³C NMR spectrum ($CDCl_3$) of **36**.

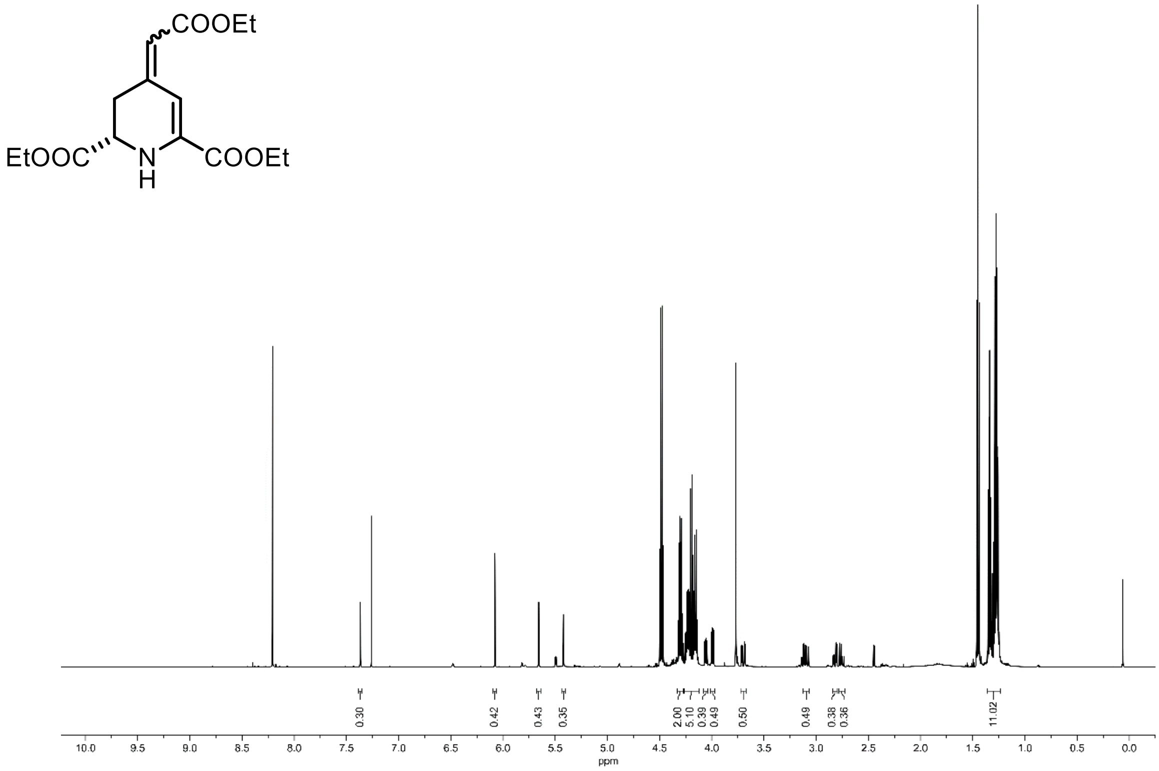

^{1}H NMR spectrum ($CDCl_3$) of **37**. The spectrum contains a mixture of *E*/*Z* isomers and its oxidized form **40**.

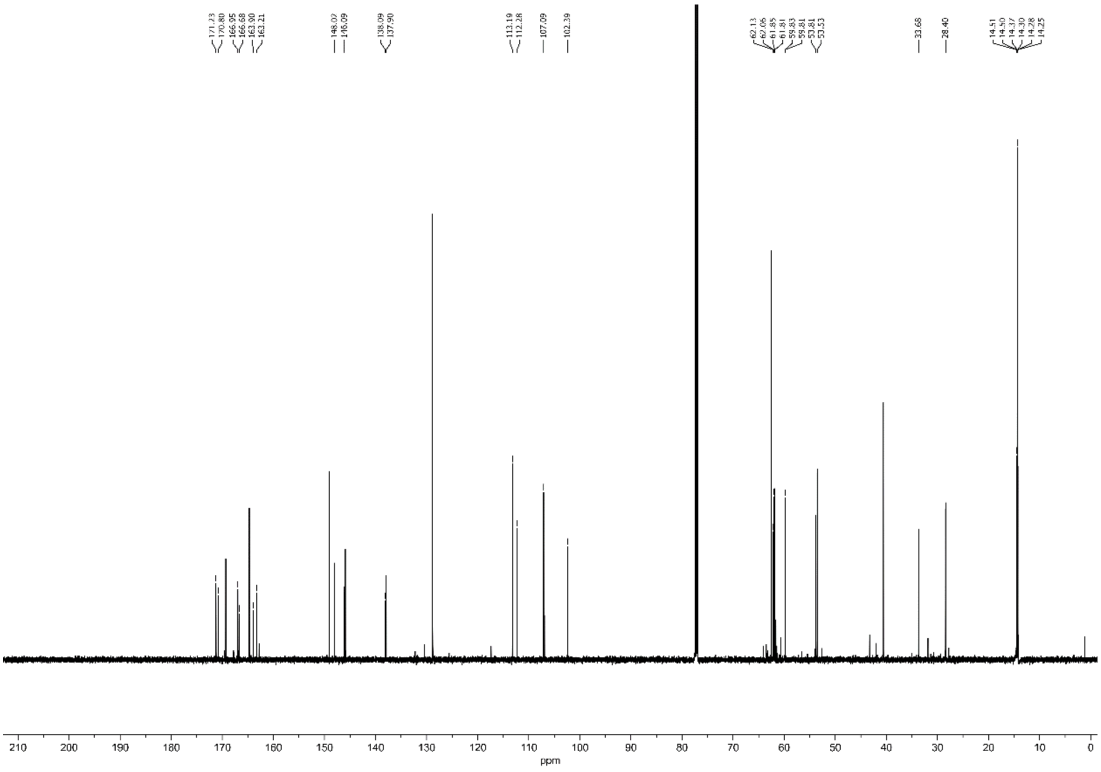

^{13}C NMR spectrum ($CDCl_3$) of **37.** The spectrum contains a mixture of *E*/*Z* isomers and its oxidized form **40**.

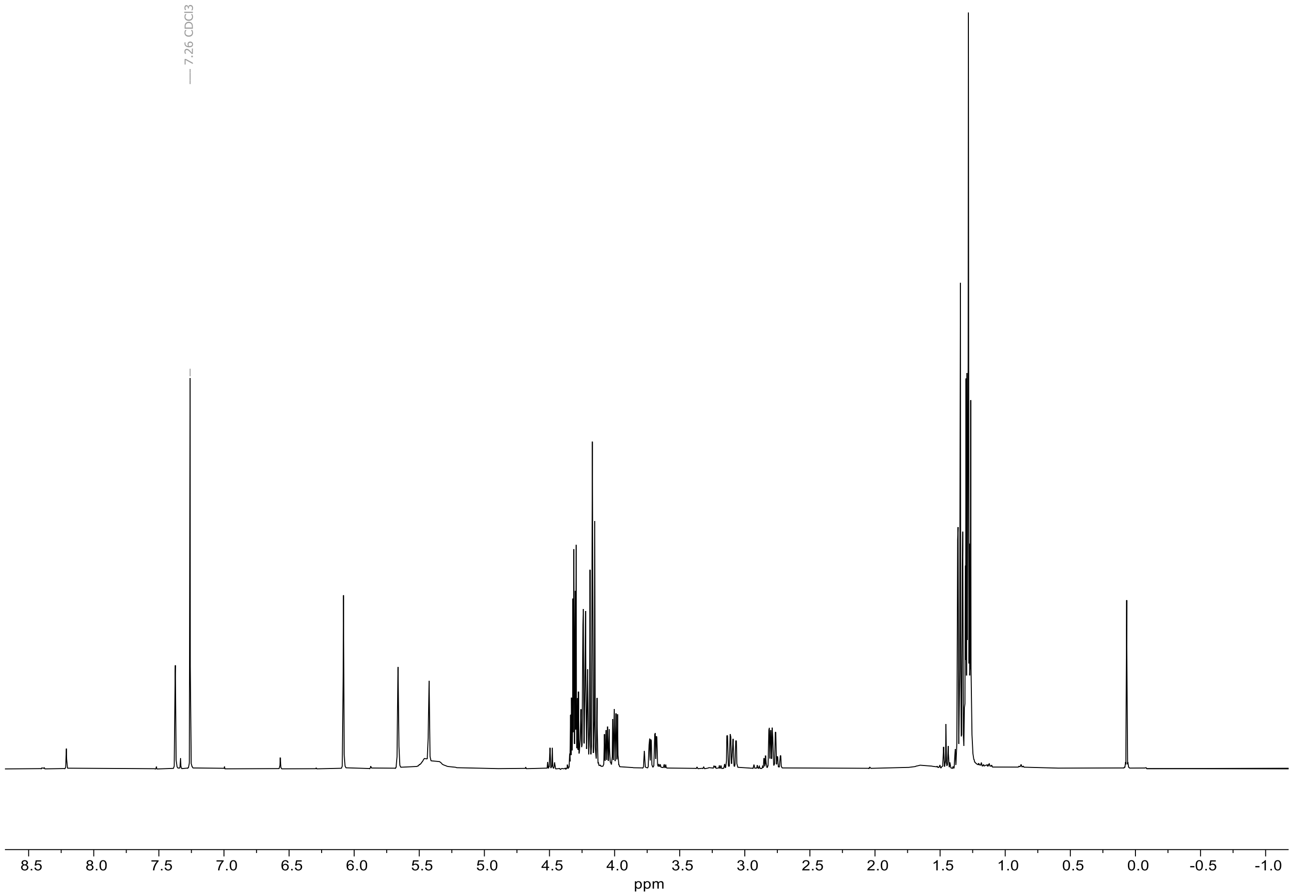

^{1}H NMR spectrum ($CDCl_3$) of **37,** 30 minutes after 2nd FC.
